# Acclimation to wind loads and/or contact stimuli? A biomechanical study of peltate leaves of *Pilea peperomioides*

**DOI:** 10.1093/jxb/erab541

**Published:** 2021-12-10

**Authors:** Max Langer, Elena Hegge, Thomas Speck, Olga Speck

**Affiliations:** 1 Plant Biomechanics Group @ Botanic Garden, University of Freiburg, Freiburg, Germany; 2 Cluster of Excellence livMatS @ FIT – Freiburg Center for Interactive Materials and Bioinspired Technologies, University of Freiburg, Freiburg, Germany; 3 University of Cambridge, UK

**Keywords:** Biomechanics, contact stimuli, factorial design, foliage leaves, thigmomorphogenesis, petiole, *Pilea peperomioides*, transition zone, wind loads

## Abstract

Plants are exposed to various environmental stresses. Leaves immediately respond to mechano-stimulation, such as wind and touch, by bending and twisting or acclimate over a longer time period by thigmomorphogenetic changes of mechanical and geometrical properties. We selected the peltate leaves of *Pilea peperomioides* for a comparative analysis of mechano-induced effects on morphology, anatomy, and biomechanics of petiole and transition zone. The plants were cultivated for 6 weeks in a phytochamber divided into four treatment groups: control (no stimulus), touch stimulus (brushing every 30 s), wind stimulus (constant air flow of 4.6 m s^−1^), and a combination of touch and wind stimuli. Comparing the four treatment groups, neither the petiole nor the transition zone showed significant thigmomorphogenetic acclimations. However, comparing the petiole and the transition zone, the elastic modulus (*E*), the torsional modulus (*G*), the *E*/*G* ratio, and the axial rigidity (*EA*) differed significantly, whereas no significant difference was found for the torsional rigidity (*GK*). The twist-to-bend ratios (*EI*/*GK*) of all petioles ranged between 4.33 and 5.99, and of all transition zones between 0.67 and 0.78. Based on the twist-to-bend ratios, we hypothesize that bending loads are accommodated by the petiole, while torsional loads are shared between the transition zone and petiole.

## Teaser

The petiole and transition zone of peltate leaves of *Pilea peperomioides*show significant differences in geometrical, mechanical, and structural properties, whereas comparatively small changes were found after wind and/or touch stimulation.

## Introduction

In their natural environment, plants are exposed to a variety of mechanical stresses such as gravity, wind, rain drops, and touch by passing animals. Since plants can perceive external mechanical stresses, mechano-stimulation induces a cascade of physiological, developmental, anatomical, and morphological processes termed thigmomorphogenesis (Jaffe, 1973; Telewski, 2021). Plants can compensate for detrimental effects of stress through immediate response, acclimation, and adaptation. Immediate response of individual plants occurs within seconds to days (Telewski, 2021). For example, the rapid wind-induced reconfiguration of plant leaves in terms of bending and twisting, which eventually results in a streamlined shape (Vogel, 1989), can prevent damage to leaves, branches, or even entire plant stems. Acclimation of individual plants to environmental constraints takes place over a time period of days and weeks (Badel *et al.*, 2015) resulting in changes of their morphological, anatomical, and mechanical properties. Such ‘mechanically trained’ plants can withstand higher environmental stresses at their individual habitats without being damaged. However, response and acclimation of individual plants should not be confused with adaptation, which is the result of genetic change in populations over an evolutionary time (Lambers *et al.*, 2008).

### Response and thigmomorphogenetic acclimation of plants

The most important mechanical environmental factor for land plants is probably wind, which bends and twists the plants and their organs (Jaffe, 1980). The large surface area of the lamina, which collects sunlight for photosynthesis, increases the contact surface for wind loads, which in turn increases the wind-induced drag and friction forces. It is part of our everyday experience that leaves immediately respond to wind gusts by fluttering followed by a reconfiguration in the wind (Vogel, 1989). Petiole and lamina streamline by bending and twisting, which markedly reduces the drag forces and thus makes higher wind loads subcritical for the leaves.

Furthermore, individual plants can also acclimate to mechanical stresses by altering their growth patterns (Lambers *et al.*, 2008). After days or weeks of mechanical perturbation, plants can change the morphological and/or mechanical properties of their organs and of single plant parts. These thigmomorphogenetic acclimations result from physiological changes in growth regulation found in trees and herbaceous plants (Telewski, 2021). Common acclimations to mechanical stimuli are reduced elongation, increased radial growth and tissue strength, premature senescence, and reduced susceptibility to various stresses like frost and drought. Jaffe (1973) termed these morphogenetic acclimations thigmomorphogenesis. The first measurable reactions to mechanical stimuli occur immediately and these acclimations can occur at the level of individual organs like leaves, parts of organs like the petioles, but also at the level of the whole plant (Jaffe, 1980; Jaffe and Forbes, 1993). Acclimation in leaves affects the surface of the lamina, which becomes smaller, and in needle-shaped leaves the elongation of the leaves is inhibited (Telewski and Jaffe, 1986; Niklas, 1991, 1996; Telewski and Pruyn, 1998). Thigmomorphogenetic changes have been studied only sporadically for petioles and are lacking for the transition zone between petiole and lamina. Jaffe (1973) and Jaffe and Forbes (1993) showed that thigmomorphogenetic acclimations depend on the plant species and also on the type and strength of the respective stimuli. For example, there is a difference in whether the plants are stimulated mechanically by an air flow or by rubbing against other plants or being touched by passing animals (Jaffe, 1973; Jaffe and Forbes, 1993). Plant axes grown under the influence of air flow can show opposite characteristics from plant axes grown under periodic purely mechanical flexing or shaking. For example, stimulation by air flow can lead to more elongated and slender plant axes instead of shorter and thicker ones as found after touch stimulation (Smith and Ennos, 2003; Anten *et al.*, 2010). This makes stimulation by wind particularly complex, as several factors overlap.

Wind can influence also physiological aspects of the plant. This is especially relevant for leaves, where it affects the leaf boundary layer and thus aspects like the gas diffusion conductivity, heat exchange rate, transpiration rate, and ultimately photosynthesis (Anten *et al.*, 2010). From a mechanical point of view, wind causes not only bending but also twisting and rubbing of the leaves against each other. Especially torque stress through twisting can cause even stronger thigmomorphogenetic acclimations compared with other mechanical stimulations (Quirk *et al.*,1975; Quirk and Freese, 1976). To understand individual aspects, such as the mechanical influence of wind, touch or a combination of both stimuli on the petiole and the transition zone of foliage leaves, a controlled factorial experimental design is necessary. Analyses of biomechanics and geometry are suitable to quantify differences between leaf parts and thigmomorphogenetic changes. A short introduction is given in the next sections.

### Biomechanics of foliage leaves

Foliage leaves usually consist of a leaf stalk (petiole), a relatively large leaf blade (lamina), and a petiole–lamina transition zone in between. In literature, the biomechanical role of the petiole is well described, especially in terms of its support for the lamina and yielding under wind loads. However, little is known about the biomechanical behaviour of the short transition zone between the rod-shaped or U-profiled petiole and the (nearly) planar lamina (Langer *et al.*, 2021b). As described above, petiole, transition zone, and lamina of foliage leaves respond to wind or to touch by passing animals by simultaneously bending downwind and twisting away. To what extent they bend and twist depends on their flexural rigidity (*EI*) and torsional rigidity (*GJ*). These structural properties combine both material properties and geometrical properties (Vogel 1992). The bending elastic modulus (*E*; SI unit: N m^−^² or Pa) and the torsional modulus (*G*; SI unit: N m^−^² or Pa) are material properties that describe the resistance to deformation in the linear-elastic range by bending and torsion, respectively. The larger the modulus, the stiffer the plant material. The axial second moment of area (*I*; SI unit: m^4^) and the polar second moment of area (*J*; SI unit: m^4^) are geometrical properties that describe the way in which small elements of an area are distributed in relation to a bending or torsional neutral plane or torsional axis, respectively. Axial and polar second moment of area can be calculated using equations depending on the geometries of the plant axes such as circle, ellipse, square, or triangle (Niklas 1992). Thus, whether a structure is stiff or flexible depends on the material and geometrical properties. To illustrate: a structure is stiff in bending if its elastic modulus *E* and axial second moment of area *I* are high, and a structure is flexible in torsion if its torsional modulus *G* and polar second moment of area *J* are low.

Because of the simultaneous bending and torsional loading of plant organs, the trade-off between their flexural and torsional rigidity is an interesting measure. Since the twist-to-bend ratio (*EI*/*GJ*) is a dimensionless variable, different plant organs can be compared. According to Niklas (1999), the twist-to-bend ratio can be adjusted either by changing the material properties or by changing the geometry or both. High twist-to-bend ratios characterize a structure that is stiff enough to prevent severe bending and flexible enough to allow (moderate) torsion. Low twist-to-bend ratios are typical for structures flexible enough to allow (moderate) bending and stiff enough to prevent severe torsion.

In fact, all literature values of the twist-to-bend ratio (*EI*/*GJ*) are calculated with the polar second moment of area (reflected in the torsional rigidity *GJ*), which is only valid for circular cross-sections and does not consider warping of the samples by torsional load. Since the polar second moment of area is the sum of the axial second moments of area in the *x*- and *y*-direction (*J*=*I*_*x*_+*I*_*y*_), the ratio *I*/*J* can never exceed 1.0 (Pasini and Mirjalili, 2006). This is different when we calculate the torsion constant *K*, sometimes also known as the torsional second moment of area. In contrast to *J* (SI unit: m^4^), the torsion constant takes into account warping of the sample through torsion. Whenever possible, it is therefore preferable to calculate the torsion constant using the equations available for this purpose (Young *et al.*, 2002) rather than the polar second moment of area. The torsion constant *K* is usually smaller than *J* (Young *et al.*, 2002), which can lead to *I*/*K*>1.0. With values above 1.0, the geometrical properties contribute to higher twist-to-bend ratios (*EI*/*GK*). Similarly, high ratios of the mechanical properties *E*/*G* contribute to high twist-to-bend ratios.

### Biomechanics of the petiole

Petioles have a high flexural rigidity, which counteracts the bending load caused by the weight of the lamina, combined with a relatively low torsional rigidity. This low torsional rigidity allows for flexible torsional behaviour of the leaves under wind loads, enabling them to adopt a streamlined configuration and thus reduce drag forces (Vogel, 1989). This interplay of rigidities is reflected in a high twist-to-bend ratio *EI*/*GJ* of the petioles. Various studies found twist-to-bend ratios of 2–100 for petioles, which means that they are 2- to 100-fold stiffer in bending than in torsion (Vogel, 1992; Ennos *et al.*, 2000).

As far as the general geometry of petioles is concerned, we mainly find circular, elliptical, and adaxially grooved cross-sections (Vogel, 1992; Ennos *et al.*, 2000; Pasini and Mirjalili, 2006; Langer *et al.*, 2021b). With the exception of a perfectly circular cross-section, the torsion constant *K* is usually smaller than *J*. In some cases, such as the U-profiled petioles of *Hosta × tardiana* ‘El Nino’ (Langer *et al.*, 2021*a*) or *Musa textiles* (Ennos *et al.*, 2000), this can lead to *I*/*K*>1.0 contributing to high twist-to-bend ratios. Flexural rigidity and twist-to-bend ratio can be increased by increasing the absolute magnitude of the axial second moment of area (Niklas, 1992). The compensation through geometrical alterations is a common concept in plants, as it is ‘cheaper’ and easier to change the geometry or shape than to invest in more or different mechanically superior materials (Wolff-Vorbeck *et al.*, 2019).

However, with the exception of U-profiled geometries, high twist-to-bend ratios of petioles are primarily the result of the material properties and thus a high *E*/*G* ratio. This conclusion is supported by the fact that plant materials are much more rigid in bending than in torsion (Niklas, 1992, 1999). In this context, we found *E*/*G* ratios of 20–60 for the petioles of four herbaceous plant species, namely *Hosta × tardiana* ‘El Nino’, *Caladium bicolor*, *Hemigraphis alternata*, and *Pilea peperomioides* (Langer *et al.*, 2021*a*). Nevertheless, plants can only build a very limited number of materials (Knippers and Speck, 2012), while they are less restricted in terms of the geometry, size, and shape of their organs or plant axes or the three-dimensional arrangement of the involved materials. Therefore, it is important to analyse both the geometrical and material properties in order to understand the structural properties of the petiole and of the petiole–lamina transition zone as a whole.

### Biomechanics of the petiole–lamina transition zone

In the literature, the transition zone between petiole and lamina is viewed or described from the scientific perspectives of systematics, morphology, anatomy, biomechanics, and biomimetics with respect to the spatial arrangement (e.g. point, area, zone, joint, juncture) or quality of connection (e.g. transition, union, attachment, junction, border) (Langer *et al.*, 2021b). However, the biomechanical behaviour of the petiole–lamina transition zone has not yet been studied in detail. It can be assumed that the transition zone is in a smooth and elastic interplay with the petiole, as tearing of the lamina from the petiole is almost never observed. This assumption is supported by the integration of multiple gradual changes of geometry, shape, size and venation within the transition zone of various leaves. With its exponential increase in the cross-sectional area and 3D arrangement of vascular tissues, the transition zone is a damage-resistant transition between petiole and lamina (Langer *et al.*, 2021b). The effect of mechano-stimulation by wind and/or touch is of particular interest as the transition zone mediates the loads between petiole and lamina (Sacher *et al.*, 2019; Wunnenberg *et al.*, 2021). Yet, the transition zone differs from the petiole mostly in its geometry, size, shape, and tissue arrangement rather than in the materials present (Langer *et al.*, 2021b).

### Aim of the study

The aim of the present study was to find answers to the following scientific question: what are the anatomical, geometrical, and biomechanical dissimilarities and similarities of petiole and petiole–lamina transition zone of the leaves of *Pilea peperomioides* in acclimation to mechanical stimuli such as wind and/or touch? We cultivated plants for 6 weeks in a phytochamber divided into four treatment groups: control (no stimulus), touch stimulus (brushing every 30 s), wind stimulus (consistent air flow of 4.6 m s^−1^), and combined touch and wind stimulus. In order to analyse both the differences between petiole and transition zone and their thigmomorphogenetic acclimations, we carried out (i) morphometric measurements, (ii) histological investigations, and (iii) torsional and tensile mechanical tests. This study offers two novelties, namely the inclusion of the petiole–lamina transition zone and the aspect of torsional behaviour.

## Materials and methods

### Plant material

**Fig. 1.**
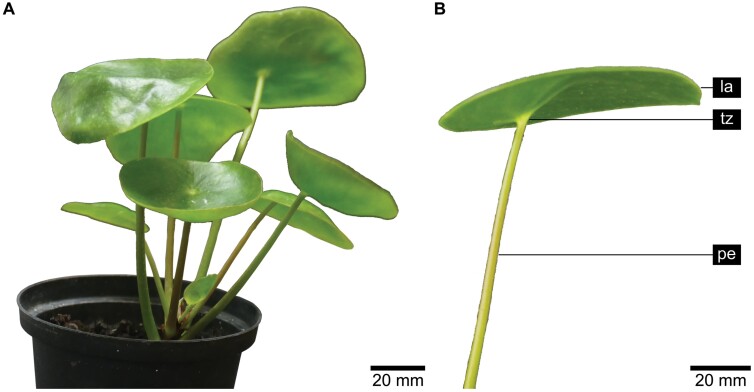
Morphology of *Pilea peperomioides* showing a whole plant (A) and a single leaf consisting of lamina (la), petiole–lamina transition zone (tz), and petiole (pe) (B).

The species *Pilea peperomioides* Diels (hereafter *P. peperomioides*) (Fig. 1) is an evergreen perennial plant that belongs to the nettle family Urticaceae. It is native only to Yunnan and Sichuan provinces in southern China and grows either in shady and damp habitats on humus-covered boulders or on cliff-ledges at altitudes of 1500–3000 m (Diels, 1909–1912; Radcliffe-Smith, 1984). *Pilea peperomioides* has orbicular peltate leaves with a rotate venation. The petiole is slightly adaxially offset and connected to the lamina at an angle of almost 90°, with a 3D-configured transition zone according to Langer *et al.* (2021b). The plants have a robust stem with the fleshy leaves being spirally arranged. The lamina has a diameter of up to 4–7 cm and the petioles are 2–17 cm long in the wild, sometimes more indoors (Wunnenberg *et al.*, 2021).

### Experimental set-up

**Fig. 2.**
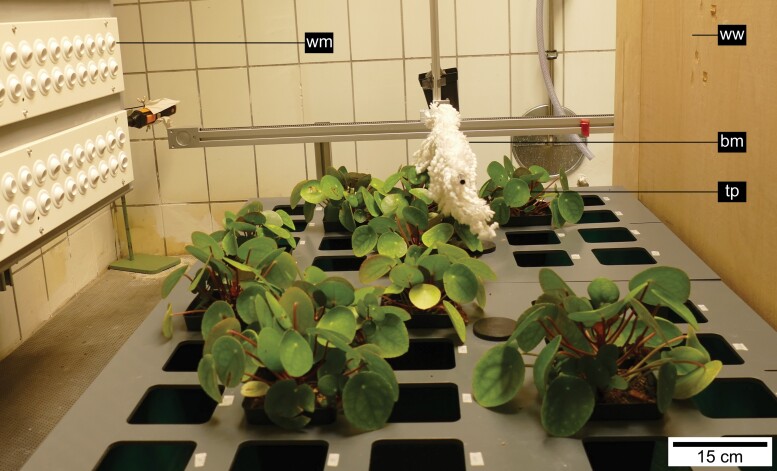
Experimental set-up. Test plants (tp) of *Pilea peperomioides* in the phytochamber under wind and touch stimulus. The wind-stimulated plants were exposed to a constant air flow of 4.6 m s^−1^ generated by the wind machine (wm). Touch-stimulated plants were brushed every 30 s by the brushing machine (bm). The plants without wind stimulus were sheltered behind the wooden windbreak (ww) visible on the right side of the picture.

Eighty individual plants of *P. peperomioides* of the same age (6 months old) were cultivated for 6 weeks in a specially designed phytochamber with an integrated wind machine (VLT® HVAC Drive FC 102 1.1–90 kW, Danfoss, Nordborg Kommune, Denmark) (Fig. 2). The average temperature in the chamber was 20 °C, the average relative humidity between 72% and 75% and the average light intensity 6086 lux with an illumination period of 12 h per day.

**Fig. 3.**
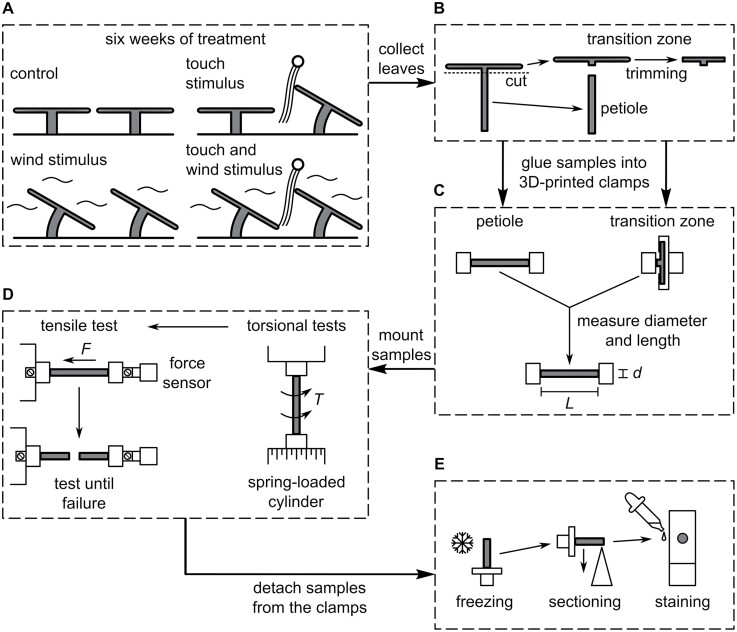
Flow chart of the experimental set-up. The plant material is highlighted in grey. (A) Treatment: the four different treatments of the plants in the phytochamber. (B) Sample preparation: separation of leaf samples into petiole and petiole–lamina transition zone. (C) Test preparation: clamping of the samples and measuring of the initial gauge length and initial diameter of the sample. (D) Mechanical tests: first torsional tests and subsequently tensile tests until failure carried out on the same sample. (E) Histological images: after sectioning of the samples and staining of the thin sections, microscopic pictures were taken.

For analysing the thigmomorphogenetic effects under wind and/or touch stimulus on biomechanics, morphology, and anatomy of the peltate leaves, a 2 × 2 factorial design was chosen as proposed by Smith and Ennos (2003) and Anten *et al.* (2010). Therefore, the plants were randomly assigned to one of four treatment groups, so that each group contained a total of 20 plants. The treatment groups consisted of a control (C) group, a touch stimulus (TS) group, a wind stimulus (WS) group, and a group with combined touch and wind stimulus (TWS) (Fig. 3A). The TS and TWS groups were touch-stimulated with a custom-built brushing machine consisting of a glass fibre rod to which microfibres of a duster were attached. Every 30 s the rod moved forward and backwards over the plant leaves. The microfibres had been attached so as to touch and stimulate the leaves without damaging the leaves or tearing them off. The WS and TWS groups were wind-stimulated by a consistent air flow averaging 4.6 m s^−1^ (Testo 400 hot wire probe, Testo, Titisee-Neustadt, Germany), generated by the wind machine integrated into the phytochamber (Supplementary Video S1). The C group remained unstimulated. Both the C and TS groups were behind a wooden windbreak, with very low air flows averaging 0.13 m s^−1^ (Fig. 2), which is considered still air according to Smith and Ennos (2003). During stimulation, each plant was rotated 90° clockwise every 2 d as gently as possible to avoid one-sided stimulation effects. Since the 20 plants in each group were arranged as carefully as possible in a 4 × 5 matrix, the plants were randomly assigned new positions in this matrix every 3 d to avoid position-dependent effects during stimulation. After 6 weeks of stimulation, the leaves of each test plant were numbered from young to old (with number 1 being the youngest leaves). One leaf of each plant numbered 8–12 with a preferably right-angled transition zone to the lamina was chosen for biomechanical testing and anatomical investigation to avoid ontogeny-dependent effects. All leaves used for testing were newly grown during the stimulation period. They were cut off with a scalpel at the base of the petiole.

### Biomechanics

For each leaf collected, the petiole was cut apically 5 mm below the lamina to obtain one petiole and one transition zone sample for each individual test plant. For the transition zone samples, the lamina part was then cut into a square of 1 cm side length around the transition zone (Fig. 3B). Both the petiole samples and the transition zone samples were glued basally and apically with an ethyl cyanoacrylate adhesive (Loctite® 401, Henkel AG & Co. KGaA, Düsseldorf, Germany) in custom-designed 3D-printed polylactide clamps. The distance between the clamps *L* was measured with a digital calliper (accuracy ±0.01 mm). In addition, the diameters in lateral direction *d*_l_ and in adaxial–abaxial direction *d*_a_ of the petiole samples were measured every 3–5 mm, depending on the petiole length, in order to obtain at least 10 measured values. Moreover, these two diameters were also measured for the middle of the petiole–lamina transition zone samples (Fig 3C).

The samples were fixed via the clamps into a custom-build manual torsional test device (Gallenmüller *et al.*, 2001), where the torque was applied by a spring-loaded cylinder (spring constant *c*=0.006 N m^−1^) and both the torque and the resulting angles were logged. Preliminary tests were carried out to ensure keeping within the linear-elastic range during the main torsion tests: the petiole samples can be twisted up to a maximum of 190°, the transition zone samples up to a maximum of 60° to 100°. Thus, in the main tests, the petiole specimens were twisted by 150°, corresponding to a median angle/length ratio of 2.3° mm^−1^, and the transition zone specimens by up to 50°, corresponding to a median angle/length ratio of 17.8° mm^−1^. This was important to avoid plastic deformation before conducting the tensile tests. After the torsional tests, the samples were mounted via the clamps in a custom-build micro tensile testing machine (Speck *et al.*, 2018), equipped with a 50 N force sensor (31E-50N0-1a-30b, Althen GmbH Mess- und Sensortechnik, Kelkheim, Germany). Tensile tests were performed with a test rate of 0.1 mm s^−1^ and the resulting forces and displacements were recorded (Fig. 3D).

As an overview, Table 1 provides a compilation and description of the material, geometrical, and structural properties measured or calculated in this study. The tapering mode is a dimensionless variable that describes the shape of a slender rod representing a circular cylinder (α=0), a second-order paraboloid of revolution (α=0.5), a circular cone (α=1), or a hyperboloid of revolution (α=1.5). Petioles possess various tapering modes (Langer *et al.*, 2021*a*). Caliaro *et al.* (2013), showed highly significant differences (*P*<0.01) when calculating the bending elastic modulus of petioles of *Caladium bicolor* with the equation considering the tapering mode compared with the equation for untapered cylindrical beams with constant circular cross-section using the mean radius of each petiole (*n*=54). Because of these significant differences, the tapering mode α will be considered in our calculations of the bending elastic modulus *E* and the torsional modulus *G*.

**Table 1.** Compilation and description of variables of material, geometrical, and structural properties, which were measured or calculated in this study

Variable	Dimension	Description
Material properties		
* E*_t_	N mm^−2^ = MPa	Elastic modulus measured in tensile tests
* G*	N mm^−2^ = MPa	Torsional modulus calculated from torsional tests
* E*/*G*	—	Calculated ratio of elastic and torsional modulus
Geometrical properties		
* A*	mm²	Measured cross-sectional area of the transverse section
* I*	mm^4^	Measured axial second moment of area of the transverse section
* K*	mm^4^	Calculated torsion constant
* I*/*K*	—	Calculated ratio of axial second moment of area and torsion constant
* *α	—	Calculated tapering mode of the petiole
Structural properties		
* EA*	N	Calculated axial rigidity
* EI*	N mm²	Calculated flexural rigidity
* GK*	N mm²	Measured torsional rigidity
* EI*/*GK*	—	Calculated ratio of flexural and torsional rigidity, also known as twist-to-bend ratio

To calculate the tapering mode α, we determined the equivalent radius *r* as follows, since tensile and not bending tests were carried out:


$$\begin{array}{l} r=\;\sqrt{r_{l}\times r_{a}}, \end{array}$$ (1)

where *r*_l_ is the radius in lateral direction and *r*_a_ the radius und adaxial–abaxial direction. According to Caliaro *et al.* (2013), the tapering mode α was calculated as follows:


$$\begin{array}{l} \alpha =\frac{log\left(\frac{r\left(x\right)-r_{apical}}{r_{basal}-r_{apical}}\right)}{log\left(\frac{L-x}{L}\right)}, \end{array}$$ (2)

where *r*(*x*) is the equivalent radius at a distance *x* from the basal petiole end, *r*_basal_ the equivalent radius at the basal petiole end (*x=*0), and *r*_apical_ the equivalent radius at the apical petiole end (*x=L*). Then the numerator was plotted against the denominator and the tapering mode α could be obtained from the slope of the linear regression.

The torsional rigidity *GK* was calculated after Gallenmüller *et al.* (2001) by:


$$\begin{array}{l} GK=\frac{L}{b_{torsion}}, \end{array}$$ (3)

with *b*_torsion_ being the slope in the angular deflection-torque diagram. The torsional modulus *G* for the transition zone samples was calculated by:


$$\begin{array}{l} G=\;\frac{GK}{K}, \end{array}$$ (4)

where *K* is the torsion constant.

The torsion constant *K* takes into account the warping of a structure that occurs under torsional loads in geometries that are not perfectly circular. Since the petioles and transition zones of *P. peperomioides* are almost circular, the following formula was used to calculate *K* (which is in this case equal to the polar axial second moment of area *J*) for solid circular sections according to Young *et al.* (2002):


$$\begin{array}{l} K=\frac{1}{2}\times \pi \times r^{4}, \end{array}$$ (5)

where *r* is the equivalent radius calculated in Eq. (1). For the petiole samples, *G* was calculated with the consideration of the tapering mode *α* as follows


$$\begin{array}{l} G=\;\frac{L}{b_{torsion}\times K_{basal}}\times {\left(\frac{r_{basal}}{r_{apical}}\right)}^{\alpha }, \end{array}$$ (6)

with *K*_basal_ being the torsion constant at the basal end of the petiole, *r*_basal_ being the radius at the basal end of the petiole, and *r*_apical_ being the radius at the apical end of the petiole.

For the tensile tests, the measured force *F* was converted into stress σ by:


$$\begin{array}{l} \sigma =\;\frac{F}{A}, \end{array}$$ (7)

where *A* is the cross-sectional area of the sample. For the tapered petioles, *A* was calculated via the axial second moment of area *I*_tapered_ that takes the tapering mode into account:


$$\begin{array}{l} I_{tapered}=I_{basal}\;\times \;{\left(\frac{r_{apical}}{r_{basal}}\right)}^{\alpha }, \end{array}$$ (8)

where *I*_basal_ is the axial second moment of area at the basal end of the petiole. Since the petioles of *P. peperomioides* are almost circular in cross-section, the cross-sectional area of the petioles *A*_tapered_, which incorporates the tapering mode, was calculated from the axial second moment of area *I*:


$$\begin{array}{l} I=\;\frac{\pi }{4}\;\times \;r^{4}, \end{array}$$ (9)

and


$$\begin{array}{l} A=\pi \;\times \;r^{2}, \end{array}$$ (10)

Therefore, if Eq. (9) is solved for the radius *r* and *I* is replaced by *I*_tapered_, we can replace the radius *r* in Eq. (10) and it follows:


$$\begin{array}{l} A_{tapered}=\pi \;\times \;{\left(\sqrt[{4}]{\frac{\;\;I_{tapered}\times 4\;}{\pi }}\right)}^{2}. \end{array}$$ (11)

After calculating the stress *σ*, the strain *ε* was computed by:


$$\begin{array}{l} \varepsilon =\;\frac{\Delta \;L}{L}, \end{array}$$ (12)

with *∆L* being the change in length. The tensile elastic modulus *E*_t_ was then calculated as the slope of the initial linear section of the stress-strain diagram:


$$\begin{array}{l} E_{t}=\;\frac{\sigma }{\varepsilon }. \end{array}$$ (13)

Thus, the axial rigidity *EA* and flexural rigidity *EI* were calculated according to


$$\begin{array}{l} EA=E_{t}\;\times \;A \end{array}$$ (14)

$$\begin{array}{l} EI=E_{t}\times \;I \end{array}$$ (15)

### Anatomy and histology

After the mechanical tests, the petiole and transition zone samples were frozen on metal sample holders by using a freezing solution (Tissue-Tek O.C.T. Compound, Sakura Finetek Japan Co., Tokyo, Japan). With a rotatory cryotome (MEV, SLEE medical, Mainz, Germany), transverse sections of 100 µm thickness were cut from the frozen samples. One section of the basal, middle, and apical petiole as well as one section of the petiole–lamina transition zone of each sample were stained with a 0.05% w/v solution of toluidine blue O for 8 min and washed in distilled water for 8 min (Fig. 3E). Toluidine blue O stains non-lignified tissue red-purple and lignified tissue blue-green (Sakai, 1973). The stained thin sections were photographed with an Olympus BX61 microscope (Olympus, Tokyo, Japan) equipped with a CP71 camera module. The cross-sectional area *A*, the axial second moment of area *I*, and the polar second moment of area *J* for each of these sections were determined numerically by using the BoneJ2 Plugin (Version 6.1.0) (Doube *et al.*, 2010) provided in the graphic software Fiji (ImageJ Version 1.52p) (Schindelin *et al.*, 2012).

For the explicit detection of lignin, additional cross-sections of the basal, middle, and apical petiole and the transition zone of each sample were stained for 1 min with a 2% w/v solution of phloroglucinol in 20% v/v ethanol in combination with a few drops of a 20% v/v solution of HCl. The phloroglucinol–HCl solution stains lignified tissue in pale pink to deep red (Gurav *et al.*, 2014). These stained cross-sections were imaged with a Zeiss Primo Star microscope (Zeiss, Jena, Germany) equipped with an Axiocam ERc 5S camera (Zeiss).

In order to obtain some highly detailed sections, one additional leaf within leaf numbers 8–12 was cut from one plant per treatment group. The leaves were fixed in formaldehyde–alcohol–acetic acid for 1 week and afterwards dehydrated via an isopropanol series (70%, 90%, 100% v/v solutions of isopropanol for 2 h each). After dehydration, the basal, middle, and apical part of the petiole and the transition zone of each leaf were embedded in Technovit® 7100 (Kulzer GmbH, Germany, Wasserburg) according to the standard embedding protocol. Thin sections with a thickness of 10 µm were prepared from the embedded samples using a rotary microtome (custom-built by the workshop, Faculty of Biology II/III, University of Freiburg). These sections were stained with a 0.05% w/v solution of toluidine blue O for 60 s and washed in distilled water for 60 s. The thin sections were recorded with an Olympus BX61 microscope equipped with a CP71 camera module.

### Statistics

All raw data are provided in the Supplementary Dataset S1. The software *GNU R* 4.0.5 was used for statistical analyses (R Core Team, 2021). The data were tested for normal distribution (Shapiro–Wilk test) and for homoscedasticity of variances (Levene’s test). Median values with corresponding interquartile ranges (IQR) are presented, since all data are non-normally distributed. We employed an α-level of 5% for the determination of significant differences. The Kruskal–Wallis test was performed together with the Mann–Whitney *U post hoc* test (with *P-*value adjustments according to Holm (1979)) for unpaired data and with Wilcoxon’s signed-rank *post hoc* test (with *P*-value adjustments according to Holm (1979)) for paired data (*P*-values are given in Supplementary Dataset S2). Levels of significance were as follows: n.s., not significant, *P*≥0.05; ∗0.05>*P*≥0.01; ∗∗0.01>*P*≥0.001; ∗∗∗0.001>*P*.

## Results

In order to analyse the various properties of the investigated petioles and petiole–lamina transition zones of the treatment groups, a large number of variables were considered. An overview of the variables is given in Table 1. Each of these variables was compared between the treatment groups of petioles and petiole–lamina transition zones (Supplementary Table S1), and between petioles and transition zones of the same treatment group (Supplementary Table S2). For ease of analysis, these variables were categorized into material properties (*E*_t_, *G*, and *E*_t_/*G)*, geometrical properties (*A*, *I*, *K*, *I*/*K*, and α) and structural properties (*EA*, *EI*, *GK*, and *EI*/*GK*).

### Material properties

**Fig. 4.**
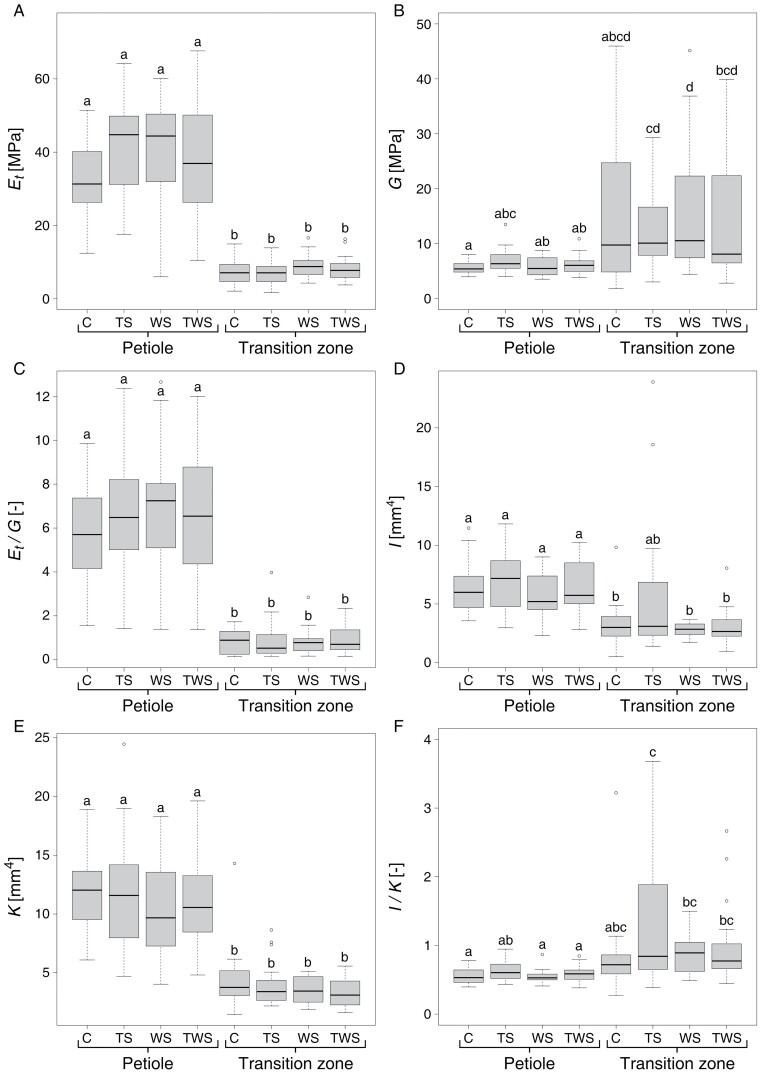
Boxplots of material properties (A–C) and geometrical properties (D–F) for each treatment group of the petiole and petiole–lamina transition zone. Each boxplot shows the median with IQR and whiskers, which indicate the outer limits of the dataset, along with the individual outliers outside these limits. (A) tensile elastic modulus *E*_t_, (B) torsional modulus *G*, (C) ratio of tensile elastic modulus and torsional modulus *E*_t_/*G*, (D) axial second moment of area *I*, (E) torsion constant *K*, (F) ratio of axial second moment of area and torsion constant *I*/*K*. The treatment groups are as follows: control (C), touch stimulus (TS), wind stimulus (WS), and touch and wind stimulus (TWS). Significant differences (*P*<0.05) are indicated by lower case letters. The sample size for the C and TS group is *n=*20 and for the WS and TWS group *n=*19.

When comparing the material properties (Fig. 4A–C) of the treatment groups of the petioles, we found no significant differences for the tensile elastic modulus *E*_t_, the torsional modulus *G*, and the ratio of tensile elastic to torsional modulus *E*_t_/*G*. This also applies when comparing the treatment groups of the transition zone. However, when comparing the material properties of the petioles and petiole–lamina transition zones of the same treatment group, *E*_t_ of the transition zone was significantly smaller than that of the petiole (Fig. 4A), whereas *G* of the transition zones was significantly higher than that of the petiole (Fig. 4B). Consequently, we found a significantly smaller *E*_t_/*G* ratio for the petiole–lamina transition zone (Fig 4C). In summary, with respect to the material properties, the petioles were stiffer in tension than in torsion, while the transition zones were stiffer in torsion than in tension (Fig. 4C).

### Geometrical properties

When comparing the geometrical properties (Fig. 4D–F) of the treatment groups of the petioles, namely the cross-sectional area *A*, the axial second moment of area *I*, the torsion constant *K*, and the ratio of axial second moment of area and torsion constant *I*/*K*, no significant differences were found. The same holds true for the geometrical properties of the different treatment groups of the petiole–lamina transition zones. When comparing the petiole–lamina transition zones and petioles of the same treatment group, we found significant differences for all but one of the geometrical properties (Supplementary Table S2). The only exception was the *I*-value of the TS group, which showed no significant difference between petiole and petiole–lamina transition zone (Fig. 4D). There was also a comparatively large variability (reflected in a higher IQR) for *I* of the petiole–lamina transition zones of the TS group compared with the transition zones of the other treatment groups. This variability was consequently also found in the corresponding *I*/*K* values of the transition zones of this treatment group (TS) (Fig. 4F). Overall, the median values of *A*, *I*, and *K* were smaller for the petiole–lamina transition zones than for the petioles (Supplementary Table S1). In contrast, the *I*/*K* ratios of the petiole–lamina transition zones were higher compared with the petioles (Fig. 4F). All petioles of all treatment groups tapered hyperbolically with α-values above 1.00. Statistical analyses showed that they do not differ significantly. In summary, geometrical properties differed significantly between petiole and petiole–lamina transition zone, but did not show significant differences with respect to the different treatments (Fig. 4D, E). The petiole–lamina transition zones had smaller median values in all studied geometrical properties and were less resistant to bending and twisting than the petioles based on their geometrical setup (Supplementary Tables S1, S2).

### Structural properties

**Fig. 5.**
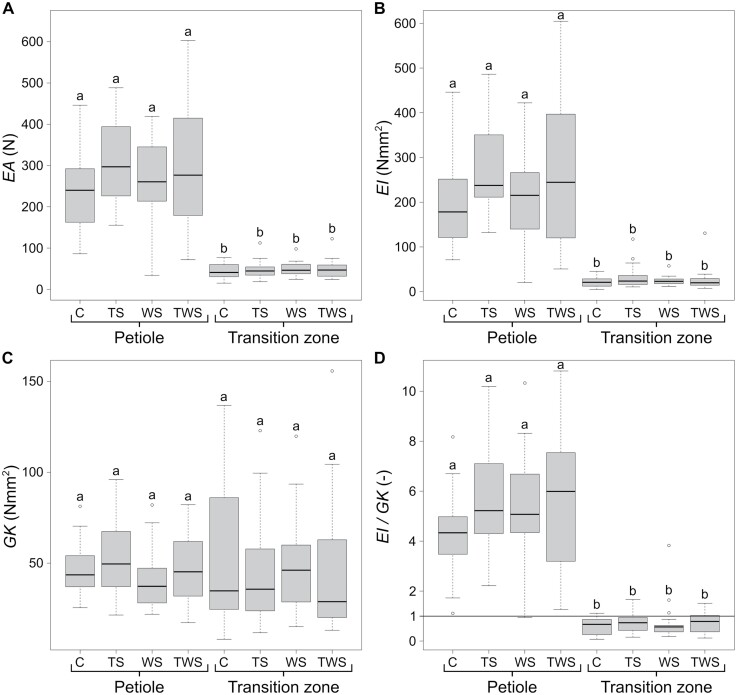
Boxplots of structural properties for each treatment group of the petiole and petiole–lamina transition zone. (A) axial rigidity *EA*, (B) flexural rigidity *EI*, (C) torsional rigidity *GK*, (D) twist-to-bend ratio *EI*/*GK*. Each boxplot shows the median with IQR and whiskers, which indicate the outer limits of the dataset, along with the individual outliers outside these limits. The treatment groups are as follows: control (C), touch stimulus (TS), wind stimulus (WS), and touch and wind stimulus (TWS). Significant differences (*P*<0.05) are indicated by lowercase letters. The sample size for the C and TS group is *n*=20 and for the WS and TWS group *n*=19.

Comparison of structural properties is provided in Fig. 5A–D. Neither for the petiole nor for the petiole–lamina transition zone were significant differences found between the treatment groups in the structural properties, such as axial rigidity *EA*, flexural rigidity *EI*, torsional rigidity *GK*, and twist-to-bend ratio *EI*/*GK*. However, when comparing petioles and petiole–lamina transition zones from the same treatment group, we found that *EA*, *EI*, and *EI*/*GK* of the petiole–lamina transition zones were significantly smaller (Supplementary Table S2; Fig. 5A, B, D). No significant differences between petiole and petiole–lamina transition zone were found for *GK* (Fig. 5C). Furthermore, the *EI*/*GK* values of the transition zones were less than one (Fig. 5D). In summary, structural properties did not differ between different treatment groups of the petioles or petiole–lamina transition zones (Fig. 5A–D). The petioles were more rigid in tension than the petiole–lamina transition zones, but did not differ significantly in torsional rigidity. The torsional rigidity of the transition zones was higher than their flexural rigidity (Supplementary Tables S1, S2).

### Anatomy

**Fig. 6.**
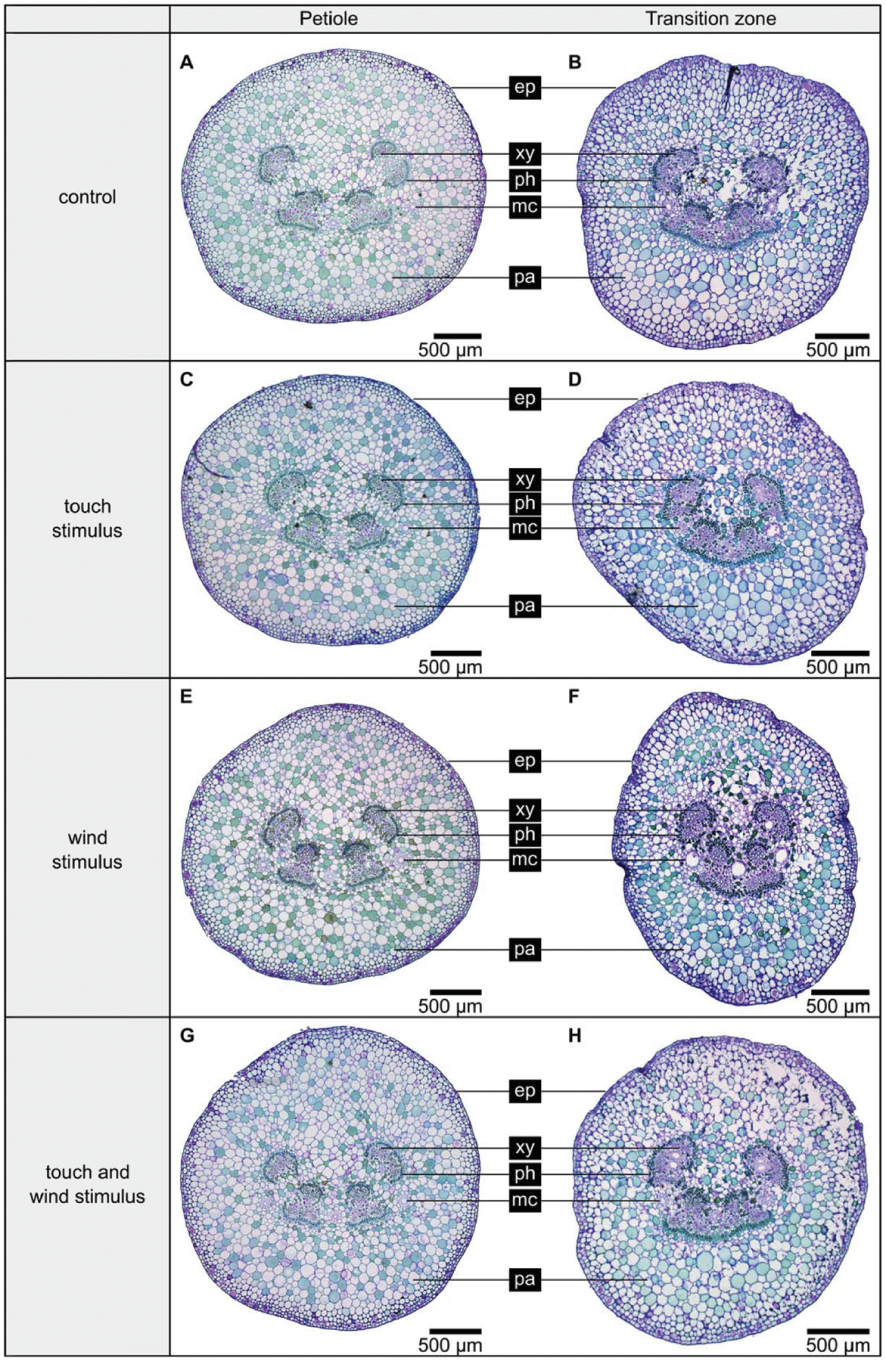
Cross-sections of the petioles and petiole–lamina transition zones of one random sample of each treatment group. The epidermis (ep), the vascular tissue (xylem [xy] and phloem [ph]), mucilage channels (mc), and parenchyma (pa) are shown. The sections are 10 µm thick and stained with toluidine blue O highlighting non-lignified tissue red-purple and lignified tissue blue-green.

When comparing the histological cross-sections of the petioles, we did not find any noticeable differences between the treatment groups, neither in the arrangement and size of the vascular tissue (xylem and phloem) (Fig. 6A, C, E, G) and mucilage channels nor in the lignification of the vascular tissue. The same applies with two exceptions to the cross-sections of the petiole–lamina transition zones comparing the treatment groups (Fig. 6B, D, F, H). The only exceptions were the mucilage channels of the transition zones, which were more pronounced in the wind-stimulated group, i.e. wind stimulus (WS). All cross-sections showed six to eight centrally arranged vascular bundles that were merged in the petioles and even more in the petiole–lamina transition zones. With the exception of the control group, the histological cross-sections of the mechano-stimulated petiole–lamina transition zones showed more or less pronounced deformations of the circular geometry. In a few sections of the transition zone of the touch stimulus group, even strong ovalization could be detected (Fig. 6). In summary, only for the petiole–lamina transition zone were minor changes in anatomy found as a result of the treatments.

## Discussion

A large number of variables that describe material, geometrical, and structural properties of peltate leaves of *P. peperomioides* were analysed to investigate acclimation of their petioles and petiole–lamina transition zones to mechanical environmental constraints such as wind stimuli, touch stimuli, or the combination of wind and touch stimuli (Table 1). We selected the species *P. peperomioides* (Fig. 1) as a model for this study because, on the one hand, we already had prior knowledge of the morphology, anatomy, and biomechanics of the petiole and the morphology and anatomy of the transition zone from previous studies (Langer et al. 2021*a*, *b*), and on the other hand, the different habitats of the plant seem to make it very suitable for studying acclimation effects, as it grows both in forests and on cliffs (Diels, 1909–1912; Radcliffe-Smith, 1984). In other words, *P. peperomioides* is native to wind-protected and open sites, which makes it likely that the fleshy leaves acclimate differently in these two habitats.

In Table 2 we have compiled the results on thigmomorphogenetic acclimation from the literature together with the results of our study. Since the mechano-stimulation of our study has focused on the primary tissues of the petiole and petiole–lamina transition zone, we limited the compilation to thigmomorphogenetic effects on primary growth. We did not include effects on secondary growth, because it is well known from literature that there are different thigmomorphogenetic effects on primary and secondary growth tissues (Paul-Victor and Rowe, 2011). For example, ethylene in response to mechanical stimulation appears to affect only secondary growth, while primary growth may not be affected (Telewski, 2021). In the following, Table 2 will serve as the basis for the discussion of the thigmomorphogenetic results found in this study.

**Table 2.** Compilation of the experimental designs and thigmomorphogenetic acclimations of material, geometrical, and structural variables of plant organs derived from the literature and this study

Species	Organ	Experimental Design	Stimulus	Material properties		Geometrical properties		Structural properties		Reference
				Variable	Change through acclimation	Variable	Change through acclimation	Variable	Change through acclimation	
*Acer saccharum*	Petiole	Field, natural wind conditions with around 6.6 m s^−1^	WS	*E*_b_	Increase (∗∗∗)	*d*	Increase (∗∗∗)	*EI*	Increase (∗∗∗)	Niklas (1996)
*Plantago major*	Petiole	Greenhouse, 46 d, constant 2.6 m s^−1^ air flow and/or brushing all 24 s	TS, WS	*E*_b_	No change	*d*	Decrease (∗∗∗)	*EI*	Decrease (∗)	Anten *et al.* (2010)
						*I*	Decrease (∗)			
			TWS		No change	*d*	No change		No change	
						*I*	No change			
*Pilea peperomioides*	Petiole	Phytochamber, 42 d, constant 4.6 m s^−1^ air flow and/or brushing all 30 s	TS	*E*_t_	Increase (n.s.)	*d*	Decrease (n.s.)	*EI*	Increase (n.s.)	This study
						*I*	Increase (n.s.)			
			WS, TWS		Increase (n.s.)	*d*	Decrease (n.s.)		Increase (n.s.)	
						*I*	Decrease (n.s.)			
*Pilea peperomioides*	Petiole–lamina transition zone	Phytochamber, 42 d, constant 4.6 m s^−1^ air flow and/or brushing all 30 s	TS	*E*_t_	No change	*d*	Decrease (n.s.)	*EI*	Increase (n.s.)	This study
						*I*	Increase (n.s.)			
			WS		Increase (n.s.)	*d*	Decrease (n.s.)		Increase (n.s.)	
						*I*	Decrease (n.s.)			
			TWS		Increase (n.s.)	*d*	Decrease (n.s.)		Decrease (n.s.)	
						*I*	Decrease (n.s.)			

For a compact overview, only studies dealing with thigmomorphogenetic changes on the primary growth tissues and with respect to bending and tension loads were considered. The stimuli are as follows: touch stimulus (TS), wind stimulus (WS), and combined touch and wind stimulus (TWS). The variables are as follows: axial second moment of area (*I*), bending elastic modulus (*E*_b_), diameter (*d*), flexural rigidity (*EI*), tensile modulus (*E*_t_). The asterisks for the significance level are assigned to the following *P*-values: n.s., not significant, *P*≥0.05; ∗0.05>*P*≥0.01; ∗∗0.01>*P*≥0.001; ∗∗∗0.001>*P*.

### General impact of the treatments

For all variables analysed in this study, although the plants were mechanically stimulated in different ways over a period of 6 weeks (Figs 2, 3), we found no significant differences between the treatment groups, neither for the petioles nor for the petiole–lamina transition zones. This holds true for the material, geometrical, and structural properties as well as for most anatomical features. Other studies also describe that plant organs—despite mechano-stimulation—show only minor or no thigmomorphogenetic changes in their investigated properties (Crook and Ennos, 1996; Anten *et al.*, 2010).

In those cases where significant thigmomorphogenetic changes affect the flexural rigidity *EI* of the stems, consisting of primary and secondary tissue, caused by wind or contact stimuli, the alterations are either a significant increase or a highly significant decrease in *EI*. In the case of the *Helianthus annuus* stem after wind stimulation and the *Arabidopsis* inflorescence stem after touch simulation, the decrease in *EI* is the result of changes in material properties, i.e. a highly significant decrease in bending elastic modulus *E*_b_, rather than changes in the geometrical properties, i.e. the diameter *d* and consequently the axial second moment of area *I* (Smith and Ennos, 2003; Paul-Victor and Rowe, 2011). In contrast, the increase in *EI* in both *Triticum aestivum* after wind stimulation and *Helianthus annuus* after touch stimulation cannot be explained by significant changes in material or geometrical properties. Rather, they are the result of different stimulation (touch stimulus (TS) for *H. annuus* and wind stimulus (WS) for *T. aestivum*) (Crook and Ennos, 1996; Smith and Ennos, 2003).

The situation is different for primary tissue organs like the petioles of *Plantago major*, where Anten *et al.* (2010) showed that the significant thigmomorphogenetic decrease of *EI* is rooted in significant alterations of the geometrical properties of the petioles. They found significantly smaller values of the diameter *d* and thus the axial second moment of area *I* in the petioles of *Plantago major* after TS and WS. In contrast, Niklas (1996) found a significant increase in *EI* by WS for the petioles of *Acer saccharum*, attributable to both a significant increase in diameter *d*, i.e. a geometrical property, and a significant increase in bending elastic modulus *E*_b_, i.e. a material property.

Unfortunately, although we have conducted an extensive literature search, we could not find any studies on the influence of touch and wind stimuli on the torsional properties of plant organs. A comparison of the thigmomorphogenetic acclimations with other plants as to these properties must therefore be omitted.

With respect to the anatomy, we found a difference in the petiole–lamina transition zones showing more pronounced mucilage channels of the WS group. Since mucilage in plants is known for its high osmotic potential, it could play a key role in the water management of *P. peperomioides* (Clifford *et al.*, 2002). Therefore, the more pronounced mucilage channels could be a thigmomorphogenetic acclimation to the higher drought stress to which the leaves are exposed by the wind.

As Lambers *et al.* (2008) described, growth experiments under controlled-environmental conditions with genetically similar plants allow an analysis of the individual effects of acclimation. For this reason, we decided on the described 2 × 2 factorial experimental design. Although our results are in good agreement with those of other studies with a similar experimental set-up, we want to discuss some reasons why no significant differences could be measured after mechanical stimulation when we compare the treatment groups of the petioles or petiole–lamina transition zones of *P. peperomioides* leaves. In general, no consensus thigmomorphogenetic acclimation emerges from literature, as it depends on the type, duration, and quality of mechanical stimulation, the selected properties measured and calculated, the investigated plant organ, and/or the respective plant species. In the case of our study on peltate leaves of *P. peperomioides*, the stimulation period might have been too short. However, care was taken to examine leaves that had newly grown during the stimulation period. Since *P. peperomioides* is a tropical evergreen plant, only neoformed leaves develop, which means that leaves develop continuously from the primordia and are not produced in a bud (Edwards *et al.*, 2016). Thus, leaf development and consequently the primary tissue of the lamina, petiole, and transition zone were likely to have been directly influenced by the various mechano-stimuli. Another explanation could be the rotation of the pots and randomization of the plant positions within their respective treatment group every few days. However, we have tried to keep the stimulation as authentic as possible, because in the field the wind blows from all directions and animals can pass along the plants from any side. Alternatively, the applied stimulations may have been in a range that the plant can compensate for without thigmomorphogenetic changes or the traits examined in our studies are ‘hard coded’ in the genome of this species and therefore do not change due to thigmomorphogenetic stimuli.

### Petioles and transition zones differ in material properties

Although very few differences were found between the treatment groups, a number of significant differences were found between the petiole and petiole–lamina transition zone. The median tensile elastic modulus *E*_t_ of the petioles is significantly higher than that of the transition zones (Fig. 4). This difference can be explained by the course and amount of the vascular bundles, the main strengthening tissue in the leaves of *P. peperomioides*. As shown by Langer *et al.* (2021b), the bundles in the petiole run perfectly straight and parallel to the longitudinal axis of the petiole, while in the transition zone the vascular bundles show an angled course towards the lamina. More importantly, the area fraction of the vascular tissue in the petiole–lamina transition zone decreases compared with the petiole, which has an impact especially under tensile loads and could cause the observed lower tensile elastic modulus *E*_t_ of the petiole–lamina transition zone. The measured *E*_t_ values of the petioles of our control plants of *P. peperomioides* range between 12 and 51 MPa. This is in good agreement with the tensile elastic moduli *E*_t_ of petioles of *Colocasia fallax* and *Tropaeolum majus*, which range between 20 and 45 MPa (Sacher *et al.*, 2019).

The bending elastic modulus of the petioles of *P. peperomioides*, however, measured in two-point bending tests, ranged between 76 and 165 MPa (Langer *et al.*, 2021*a*). To investigate this difference between the bending and tensile elastic moduli, verification measurements were made with 10 petioles of *P. peperomioides* (Supplementary Dataset S3). Five of these petioles were from younger leaves like those investigated in the present study and five were from older leaves like those investigated by Langer *et al.* (2021*a*). Each petiole was first tested in the linear-elastic range under bending load (two-point bending tests) and then under tensile load until failure. Both the bending and tensile elastic moduli were then calculated. The age of the leaves had no significant influence on either the bending or the tensile elastic modulus. However, the tensile elastic modulus was significantly smaller than the bending elastic modulus. The median value of the bending elastic moduli of the verification measurements was 127 MPa, which is in good agreement with the bending elastic moduli measured by Langer *et al.* (2021*a*). The median value of the tensile elastic moduli obtained from the verification measurements was 49 MPa, which is consistent with the tensile elastic moduli measured in this study. The difference between the tensile elastic moduli and the bending elastic moduli is therefore probably attributable to the anatomical inhomogeneity (constructed of different materials) and the mechanical anisotropy (different mechanical properties in the main directions) of the biological samples. While in tension it is mainly important which and how much of the respective materials are present in the cross-section of the structure (and their arrangement is of minor importance), in bending it is also crucial where and how these materials are arranged in the cross-section (Wolff-Vorbeck *et al.*, 2021). Under tensile loading, only tensile stresses act rather uniformly across the cross-section. In contrast, under bending loading, both tensile and compressive bending stresses act, which reach their highest intensity at the periphery of the cross-section (Niklas, 1999). This is likely to result in higher bending elastic moduli in the leaves of *P. peperomioides* compared with its tensile elastic moduli.

The torsional modulus *G* of the petiole–lamina transition zone of *P. peperomioides* is significantly higher than the torsional modulus of the petiole. This result most probably is related to the three-dimensional arrangement of the vascular bundles. As shown in Fig. 6, the bundles are clustered in the centre of the cross-sections, both in the petiole and in the petiole–lamina transition zone. In the petiole, the six to eight bundles are present separately, while in the transition zone they are merged until they split in all directions at the apical end toward the lamina (Langer *et al.*, 2021b). Structures consisting of individual separate bundles are known to be easily twisted (Wolff-Vorbeck *et al.*, 2019). The merged bundles in the transition zone, however, form an almost closed circle, which is known to be markedly more resistant to torsion (Niklas, 1999), thus providing an explanation for the higher *G* of the transition zone.

The significantly higher *E*_t_ values of the petioles and the significantly higher *G* values of the petiole–lamina transition zone indicate that the petiole and transition zone are structures ‘optimized’ to withstand different mechanical loads. In terms of material properties, the petiole can better cope with bending and tensile loads, while the transition zone can better cope with torsional loads. The differences in *E*_t_ and *G* between petiole and petiole–lamina transition zone also result in a significantly higher *E*_t_/*G* ratio of the petioles, compared with the transition zone. This in turn results in the higher twist-to-bend ratios *EI*/*GK* of the petioles.

### Petioles and transition zones differ in geometrical properties

In terms of geometrical properties, we found only a few differences between the treatment groups, but again significant differences between the petioles and petiole–lamina transition zones. Overall, the transition zone exhibits smaller values than the petiole in all geometrical properties. Since we found almost circle-like geometries for the petiole–lamina transition zone and for the petiole, the *I*/*J* ratios for both were in the region of 0.5. However, biological structures are never perfectly circular in cross-sectional geometry. Consequently, the torsion constant *K* is less than *J* for both the petiole–lamina transition zones and the petioles. For the petioles, *K* is 16–25% smaller than *J*, for the transition zone 35–48%. The reason for the deviation of the *K* values from the *J* values is the ovalization of the cross-sections, which is more pronounced in the petiole–lamina transition zone than in the petioles (Fig. 6). The lower *K* values of the petiole–lamina transition zones lead to the significantly higher *I*/*K* ratios compared with the petioles (Fig. 4). These higher *I*/*K* ratios in turn mean that the geometry of the petiole–lamina transition zone reduces the twist-to-bend ratio less than the geometry of the petiole. The calculated *I*/*J* ratios correspond to the *I*/*J* ratios of petioles and petiole–lamina transition zones of not mechanically treated *P. peperomioides* plants reported by Langer *et al.* (2021b).

### Connecting the pieces

When comparing the mechanical performance of petioles and petiole–lamina transition zones, it is noticeable that—regardless of the respective mechanical treatment—they differ significantly in axial rigidity *EA* and flexural rigidity *EI*, while torsional rigidity *GK* is not significantly different (Fig. 5; Supplementary Table S2). Each of the above-mentioned rigidities is the product of a mechanical material property (*E* or *G*) and a geometrical property (*A*, *I*, or *K*) of the overall structure. In the literature, the twist-to-bend ratio (*EI*/*GK)* is a measure of the trade-off between flexural and torsional rigidity with the great advantage of being dimensionless for comparisons with other treatments and/or plant organs.

In this study, we could not perform reliable bending tests with the very short petiole–lamina transition zones. In order to compare the values between transition zone and petiole, we performed tensile tests with all specimens with the limitation that we can only calculate the tensile elastic modulus. Thus, we had to calculate the flexural rigidity from the tensile elastic modulus *E*_t_, the latter being smaller than the bending elastic modulus *E*_b_. Since we calculate the flexural rigidity using the tensile elastic modulus *E*_t_, it is reasonable to assume that we underestimate the flexural rigidity calculated in this study compared with the flexural rigidities determined by bending tests. Consequently, the twist-to-bend ratios are also underestimated, which must be taken into account when making comparisons with twist-to-bend ratios in other studies. Only Louf *et al.* (2018) also calculate the twist-to-bend ratios of petioles based on tensile and torsion tests. To calculate the flexural rigidity, they used the tensile elastic modulus, and to calculate the torsional rigidity, they used the polar second moment of area. They studied the petioles of four tree species exhibiting average twist-to-bend ratios between 3.0 and 6.3, which are in good agreement with our median values of the petioles of all treatment groups.

Knowing that *GK* of the petioles and petiole–lamina transition zones of *P. peperomioides* is not significantly different, high twist-to-bend ratios of petioles must arise from high *EI* and low twist-to-bend ratios of the transition zones must arise from low *EI*. Twist-to-bend ratios of the transition zones even fall below 1.0 because the torsional rigidity is higher than the flexural rigidity. Alternatively, the twist-to-bend ratio can be considered from the viewpoint of the material properties: the ratio of the geometrical properties *I*/*K* is mostly below 1.0 and, thus, does not contribute to high but to low twist-to-bend ratios. The ratio of the mechanical properties *E*/*G* can take values far above 1.0 in plants and, thus, contributes markedly to high twist-to-bend ratios.

Overall, the high *EI* values of the petiole and the similar *GK* values translate into the significantly higher twist-to-bend ratios *EI*/*GK* compared with the petiole–lamina transition zone. The twist-to-bend ratio of the petiole of *P. peperomioides* found in this study is quite in line with that of other petioles (Vogel, 1992; Ennos *et al.*, 2000; Louf *et al.*, 2018). The petiole–lamina transition zone, however, has twist-to-bend ratios of less than 1. To our knowledge, such a low value has not yet been measured for any biological structure. Based on the material, geometrical, and structural properties, we hypothesize an allocation of the biomechanical functions between the petiole and petiole–lamina transition zone. The hypothesis is supported by the findings of Niklas (1999), who reported that the maximum tensile stresses through bending occur at the base of the petiole. This is consistent with the result of the significantly higher flexural rigidity *EI* of the petiole of *P. peperomioides* compared with the petiole–lamina transition zone, in terms of both the material and the geometry. Niklas (1999) additionally described that the maximum torsional shear stresses in the petioles occur directly below their point of attachment to the lamina, which corresponds exactly to the petiole–lamina transition zone (Langer *et al.*, 2021b). Our results show, however, that the transition zone and the petioles have similar torsional rigidity *GK*, and thus they seem to share the torsional loads. This, though, is not at all in contradiction to Niklas (1999), but instead supports his findings. Based on his classical mechanical theory, the torsional load in the transition zone would have to be even higher than at the apical end of the petiole. Therefore, the mechanism of torsional load sharing between the two structures is reasonable. This spatial allocation of bending and torsional loads between the petiole and the transition zone explains the resistance of the petiole–lamina transition zone and the fact that damage is rarely found there.

The significantly higher elastic modulus *E*_t_ of the petiole in combination with the likewise higher cross-sectional area *A* and axial second moment of area *I* leads to the higher axial rigidity *EA* and flexural rigidity *EI* compared with the petiole–lamina transition zone (Supplementary Table S2). Although we tend to underestimate flexural rigidity of the petioles by using the tensile elastic modulus for the calculation, the *EI* values of *P. peperomioides* are in the same range as those measured by Anten *et al.* (2010) for the petioles of *Plantago major*.

In summary, the petioles are better equipped for tensile and bending loads, in terms of both material and geometrical properties. In contrast, the petiole–lamina transition zones can better cope with torsional loads, which is mirrored by their significantly higher torsional modulus *G*. These higher *G* values of the petiole–lamina transition zone, however, are counterbalanced by lower torsion constants *K* compared with the petiole. Due to the higher torsional modulus and the lower torsion constant of the transition zones, similar torsional rigidities *GK* are found for the petioles and petiole–lamina transition zones.

## Conclusion

As the transition zone is an often overlooked part of the leaf, only very few morphological, anatomical, and biomechanical studies or comparative analyses with the petiole can be found in the literature. Equally, thigmomorphogenetic changes have been studied only sporadically for petioles and are lacking for the transition zone between petiole and lamina.

With respect to our scientific question, ‘What are the anatomical, geometrical, and biomechanical dissimilarities and similarities of petiole and petiole–lamina transition zone of the leaves of *Pilea peperomioides* in acclimation to mechanical stimuli such as wind and/or touch?’, we can provide some answers. A comparison with the literature on acclimation shows that our study is the first to investigate torsional behaviour and thus the twist-to-bend ratio could be analysed. Moreover, for the first time we distinguished mechanically between the petiole and the petiole–lamina transition zone of a foliage leaf.

We can state that elastic modulus, torsional modulus, ratio of elastic and torsional modulus, and axial rigidity of petioles and petiole–lamina transition zones of the peltate leaves of *P. peperomioides* differed significantly. In contrast, the torsional rigidity of petioles and transition zones did not differ significantly. On the one hand, the flexural rigidity of petioles was four to six times higher than the torsional rigidity, resulting in twist-to-bend ratios of 4.0 and higher. On the other hand, the torsional rigidity of the petiole–lamina transition zones was comparatively higher than their flexural rigidity, leading to twist-to-bend ratios of below 1.0. The mechano-stimulation did not generate significant biomechanical differences between the different treatment groups. This holds true for the petioles and for the petiole–lamina transition zones. Only for the petiole–lamina transition zone, minor mechano-induced changes in anatomy in the form of more pronounced mucilage channels were found. Our findings on acclimation of the leaves of *P. peperomioides* therefore confirm the results of other studies showing that acclimation is a very species-dependent trait. On the basis of the twist-to-bend ratios of 4.0 and more, we hypothesize that bending loads are accommodated by the petiole. Moreover, based on comparable values of torsional rigidity, we hypothesize that the torsional loads are shared between the transition zone and the petiole.

Our study provides new insights into the mechanical functioning of foliage leaves, and also into the general interaction of plants with complex mechanical environmental loads such as wind stimulus, touch stimulus, or a combination of both stimuli. It would be very interesting to study the mechanical, geometrical, and structural properties of petioles and transition zones of other foliage leaves. Foliage leaves differ in their general body structure (mono- or dicotyledons) and their spatial arrangement of petiole and lamina. Peltate leaves are characterized by a 3D arrangement where the petiole and the lamina have approximately an angle of 90°. In foliage leaves with a 2D arrangement, the petiole is directly attached to the margin of the lamina. Comparing various foliage leaves being exposed to wind, touch, or a combination of both stimuli should be discussed in a broader ecological and ecophysiological context, which would help to better understand the complex interaction of one of the plants most essential organs with the environment.

## Supplementary data

The following supplementary data are available at *JXB online*.

Dataset S1. Raw data of the various experiments carried out.

Dataset S2. Results (*P*-values) of the different statistical tests performed.

Dataset S3. Verification measurements of the bending and tensile elastic moduli.

Table S1. Descriptive statistics of the variables measured and calculated for the leaf parts of *Pilea peperomioides*.

Table S2. Ratios of median values of petiole–lamina transition zone to petiole for each variable and for each treatment group.

Video S1. Video of the experimental cultivation setup in the phytochamber.

erab541_suppl_supplementary_datasets_S1-S3

erab541_suppl_supplementary_tables_S1-S2

erab541_suppl_supplementary_video_S1

## Data Availability

All data supporting the findings of this study are available within the paper and within its supplementary materials published online.
