# Supplementary material for: Acclimation to wind loads and/or contact stimuli? A biomechanical study of peltate leaves of Pilea peperomioides
Source: J Exp Bot. 2021 Dec 10;73(4):1236–52. doi: 10.1093/jxb/erab541 (PMC8866637; doi:10.1093/jxb/erab541)
Supplement: erab541_suppl_supplementary_datasets_S1-S3 [file erab541_suppl_supplementary_datasets_s1-s3.pdf]

Sample	Control group (C)		Touch stimulus group (TS)	
	Petiole	Transition zone	Petiole	Transition zone
	[MPa]	[MPa]	[MPa]	[MPa]
1	33.00	3.83	31.32	4.40
2	46.69	6.09	20.34	3.97
3	36.35	9.65	58.38	1.66
4	41.96	12.97	49.16	11.65
5	38.32	6.56	17.57	3.83
6	12.67	7.41	50.00	4.91
7	27.69	9.93	35.25	6.13
8	48.29	13.52	64.15	9.18
9	12.40	2.06	49.54	7.36
10	34.51	7.15	56.09	4.92
11	14.80	6.98	49.67	3.93
12	26.42	2.98	41.59	8.10
13	14.99	4.62	58.46	8.36
14	27.98	7.19	48.70	8.46
15	51.44	9.10	31.03	7.49
16	29.60	4.71	44.92	6.74
17	33.74	14.93	41.78	6.57
18	28.89	3.93	19.20	12.08
19	42.33	8.32	21.22	13.86
20	26.20	4.82	44.59	10.06
**Median**	31.30	7.07	44.75	7.05
**IQR**	12.86	4.55	18.51	3.86

Sample	Wind stimulus group (WS)		Touch and wind stimulus group (TWS)	
	Petiole	Transition zone	Petiole	Transition zone
	[MPa]	[MPa]	[MPa]	[MPa]
1	50.53	14.14	39.38	5.08
2	56.81	7.10	26.49	5.33
3	44.84	5.54	10.41	8.14
4	49.54	8.97	47.19	6.88
5	50.30	9.35	19.10	7.09
6	5.97	9.32	31.77	16.24
7	21.95	10.90	24.29	6.07
8	16.18	10.77	66.59	6.65
9	17.82	5.00	36.91	7.71
10	60.11	16.60	45.25	9.37
11	34.81	5.47	60.93	8.59
12	41.71	7.63	67.64	4.83
13	44.39	7.20	65.56	11.32
14	44.49	7.39	29.46	9.72
15	50.47	10.07	47.63	5.51
16	32.17	8.75	26.10	15.50
17	51.48	12.12	15.07	11.55
18	35.36	4.25	36.05	3.74
19	31.70	6.07	52.60	9.56

**Median**	44.39	8.75	36.91	7.71
**IQR**	18.45	3.83	23.83	3.85

Sample	Control group (C)		Touch stimulus group (TS)	
	**Petiole**	**Transition zone**	**Petiole**	**Transition zone**
	[MPa]	[MPa]	[MPa]	[MPa]
1	4.85	27.70	4.46	17.14
2	6.13	19.99	6.10	22.41
3	6.40	45.98	5.16	10.79
4	6.24	9.63	9.73	5.35
5	5.39	7.01	7.92	8.53
6	4.52	5.70	9.07	9.77
7	6.40	38.99	6.66	11.14
8	5.65	22.88	8.07	7.25
9	7.94	1.85	5.85	29.30
10	3.98	4.14	5.94	16.18
11	8.02	29.66	4.02	10.43
12	6.62	2.84	7.99	27.69
13	4.46	21.45	5.20	13.32
14	4.89	5.57	6.26	9.06
15	5.22	22.01	9.76	6.28
16	5.39	3.14	6.42	18.55
17	6.65	9.16	6.30	6.46
18	4.69	26.58	13.49	3.04
19	4.84	9.89	4.26	8.68
20	5.43	3.95	7.05	9.18
**Median**	5.41	9.76	6.36	10.10
**IQR**	1.55	18.59	2.32	8.20

Sample	Wind stimulus group (WS)		Touch and wind stimulus group (TWS)	
	**Petiole**	**Transition zone**	**Petiole**	**Transition zone**
	[MPa]	[MPa]	[MPa]	[MPa]
1	7.47	36.88	6.47	32.54
2	7.77	14.91	3.86	5.20
3	3.54	6.44	4.45	32.00
4	6.64	23.42	8.58	6.39
5	8.75	5.94	4.85	5.25
6	4.29	21.22	5.62	34.57
7	5.32	14.47	6.86	8.64
8	6.17	9.67	6.20	39.89
9	4.72	30.19	4.50	17.34
10	7.40	5.84	6.92	19.66
11	7.82	6.22	5.34	8.10
12	5.25	45.15	6.06	6.88
13	3.76	9.23	8.78	7.53
14	4.08	24.22	6.15	6.61
15	7.94	10.54	5.09	2.82
16	4.08	8.42	7.28	25.10
17	6.04	12.65	10.90	4.94
18	5.49	4.44	5.12	13.69
19	4.38	10.50	4.38	6.98

**Median**	5.49	10.54	6.06	8.10
**IQR**	3.10	14.89	1.92	15.88

Ratios of tensile elastic moduli to torsional moduli E_t/G

Sample	Control group (C)		Touch stimulus group (TS)	
	Petiole	Transition zone	Petiole	Transition zone
	[-]	[-]	[-]	[-]
1	6.80	0.14	7.02	0.26
2	7.62	0.30	3.34	0.18
3	5.68	0.21	11.31	0.15
4	6.72	1.35	5.05	2.18
5	7.11	0.94	2.22	0.45
6	2.80	1.30	5.51	0.50
7	4.33	0.25	5.29	0.55
8	8.55	0.59	7.95	1.27
9	1.56	1.11	8.47	0.25
10	8.67	1.73	9.44	0.30
11	1.84	0.24	12.36	0.38
12	3.99	1.05	5.21	0.29
13	3.36	0.22	11.25	0.63
14	5.72	1.29	7.78	0.93
15	9.85	0.41	3.18	1.19
16	5.49	1.50	7.00	0.36
17	5.08	1.63	6.64	1.02
18	6.15	0.15	1.42	3.98
19	8.75	0.84	4.98	1.60
20	4.83	1.22	6.33	1.10
**Median**	5.70	0.89	6.48	0.53
**IQR**	3.00	1.04	3.05	0.82

Sample	Wind stimulus group (WS)		Touch and wind stimulus group (TWS)	
	Petiole	Transition zone	Petiole	Transition zone
	[-]	[-]	[-]	[-]
1	6.77	0.38	6.08	0.16
2	7.31	0.48	6.86	1.03
3	12.65	0.86	2.34	0.25
4	7.46	0.38	5.50	1.08
5	5.75	1.57	3.94	1.35
6	1.39	0.44	5.66	0.47
7	4.13	0.75	3.54	0.70
8	2.62	1.11	10.74	0.17
9	3.78	0.17	8.20	0.44
10	8.12	2.84	6.54	0.48
11	4.45	0.88	11.41	1.06
12	7.94	0.17	11.16	0.70
13	11.82	0.78	7.47	1.50
14	10.91	0.31	4.79	1.47
15	6.36	0.95	9.36	1.95
16	7.89	1.04	3.59	0.62
17	8.53	0.96	1.38	2.34
18	6.44	0.96	7.04	0.27
19	7.24	0.58	12.00	1.37

**Median**	7.24	0.78	6.54	0.70
**IQR**	2.93	0.55	4.41	0.90

Sample	Control group (C)		Touch stimulus group (TS)	
	Petiole	Transition zone	Petiole	Transition zone
	[mm^2]	[mm^2]	[mm^2]	[mm^2]
1	7.78	5.03	9.47	5.11
2	10.08	5.98	11.51	8.59
3	7.58	2.96	8.60	11.17
4	8.00	5.38	7.81	4.60
5	8.22	6.18	10.83	5.94
6	9.03	4.89	7.13	5.55
7	10.58	7.13	9.05	5.69
8	9.73	5.29	9.80	5.49
9	9.83	7.52	8.39	4.82
10	10.89	6.03	11.88	13.64
11	12.19	6.71	7.30	9.56
12	7.81	5.03	12.58	8.97
13	9.33	8.96	9.36	6.46
14	9.76	7.47	9.89	6.44
15	10.19	7.30	10.75	6.63
16	9.23	6.90	8.86	5.49
17	10.29	5.15	10.38	6.94
18	11.85	5.28	11.81	6.22
19	9.52	5.69	14.32	8.12
20	8.53	7.15	6.91	4.32
**Median**	9.62	6.01	9.64	6.33
**IQR**	1.77	1.89	2.45	2.74

Sample	Wind stimulus group (WS)		Touch and wind stimulus group (TWS)	
	Petiole	Transition zone	Petiole	Transition zone
	[mm^2]	[mm^2]	[mm^2]	[mm^2]
1	5.75	4.84	8.36	4.79
2	6.78	6.58	9.48	6.01
3	9.00	5.88	8.05	6.05
4	12.47	6.26	10.25	5.83
5	10.40	6.98	8.26	5.11
6	7.34	4.97	10.19	7.55
7	8.56	5.55	7.97	5.24
8	8.83	5.98	11.77	7.04
9	10.40	5.81	6.56	3.74
10	8.28	5.89	11.03	6.35
11	10.99	6.89	11.51	6.14
12	6.47	5.53	8.80	5.40
13	9.43	5.95	8.80	5.62
14	8.13	5.12	8.77	5.27
15	8.27	5.74	11.11	5.99
16	8.12	5.29	8.72	3.78
17	8.51	5.03	9.55	6.52
18	7.88	5.73	8.75	6.43
19	10.46	6.18	11.46	7.13

**Median**	8.51	5.81	8.80	5.99
**IQR**	1.91	0.67	2.09	1.13

Sample	Control group (C)		Touch stimulus group (TS)	
	**Petiole**	**Transition zone**	**Petiole**	**Transition zone**
	[mm^4]	[mm^4]	[mm^4]	[mm^4]
1	3.56	2.33	7.50	2.41
2	6.26	3.13	11.82	9.73
3	4.08	0.51	5.55	18.57
4	4.59	2.19	4.47	1.67
5	4.55	2.98	8.70	3.28
6	5.62	2.09	3.95	2.26
7	8.21	3.35	6.46	2.70
8	5.82	2.37	6.77	2.26
9	7.26	3.88	4.87	2.09
10	7.46	3.19	8.67	23.91
11	10.42	3.02	3.39	9.67
12	3.90	2.05	11.01	9.07
13	6.39	9.83	6.89	2.95
14	5.68	4.00	7.45	3.12
15	8.67	4.86	8.44	3.89
16	6.16	4.43	4.68	3.07
17	7.12	1.66	8.10	3.46
18	11.46	2.42	11.07	2.85
19	5.81	2.72	10.78	4.62
20	4.77	4.12	2.97	1.39
**Median**	5.99	3.00	7.17	3.09
**IQR**	2.59	1.62	3.85	3.36

Sample	Wind stimulus group (WS)		Touch and wind stimulus group (TWS)	
	**Petiole**	**Transition zone**	**Petiole**	**Transition zone**
	[mm^4]	[mm^4]	[mm^4]	[mm^4]
1	2.31	1.73	6.21	1.76
2	3.53	3.67	5.40	2.65
3	5.43	2.41	4.88	3.71
4	8.52	3.18	8.86	2.61
5	7.50	3.68	4.67	2.19
6	3.47	2.33	8.14	8.05
7	5.45	3.19	4.78	2.18
8	4.79	2.42	9.08	4.38
9	9.00	3.39	2.84	0.98
10	4.36	3.47	7.35	3.12
11	8.65	3.64	8.87	3.14
12	2.67	2.84	5.15	2.34
13	7.26	2.38	5.73	2.31
14	4.84	2.16	5.46	2.39
15	4.67	2.90	10.23	3.60
16	5.30	2.64	4.77	0.95
17	5.20	2.32	6.28	3.06
18	4.98	2.81	5.64	4.75
19	8.34	3.06	9.47	4.10

**Median**	5.20	2.84	5.73	2.65
**IQR**	2.87	0.89	3.49	1.40

Sample	Control group (C)		Touch stimulus group (TS)	
	Petiole	Transition zone	Petiole	Transition zone
	[mm^4]	[mm^4]	[mm^4]	[mm^4]
1	6.84	2.36	8.55	2.22
2	15.83	4.65	13.19	2.64
3	6.30	1.45	12.86	3.09
4	8.40	3.05	7.05	2.19
5	10.25	4.13	11.78	3.39
6	13.91	3.54	6.71	2.81
7	10.82	3.51	9.48	5.05
8	13.34	5.09	15.24	3.08
9	13.12	14.30	8.22	3.40
10	18.89	5.50	17.10	7.59
11	13.38	2.67	6.38	2.27
12	6.09	2.87	15.18	3.48
13	12.33	3.05	11.47	4.02
14	11.88	5.24	12.60	4.70
15	15.14	5.99	19.00	3.80
16	12.18	6.16	7.72	2.65
17	10.74	3.86	11.13	3.94
18	15.02	3.65	11.69	7.39
19	11.18	3.45	24.46	8.64
20	8.81	4.52	4.70	2.18
**Median**	12.03	3.76	11.58	3.39
**IQR**	3.63	2.08	5.60	1.54

Sample	Wind stimulus group (WS)		Touch and wind stimulus group (TWS)	
	Petiole	Transition zone	Petiole	Transition zone
	[mm^4]	[mm^4]	[mm^4]	[mm^4]
1	4.02	1.88	7.34	2.27
2	6.98	4.12	11.32	3.64
3	13.26	3.44	8.14	2.25
4	14.42	5.11	11.36	3.10
5	15.64	3.53	8.93	3.34
6	6.86	2.22	10.42	3.02
7	6.28	2.13	12.50	2.36
8	9.74	4.77	18.44	3.90
9	13.93	3.10	4.81	1.60
10	7.57	2.58	13.13	4.69
11	18.28	4.99	16.22	4.10
12	5.58	2.00	8.79	1.90
13	12.51	4.87	13.39	3.06
14	9.16	2.42	10.16	4.49
15	9.20	5.13	15.12	4.66
16	9.06	3.15	7.87	2.14
17	9.67	3.74	10.56	5.57
18	9.69	4.55	7.12	2.10
19	13.85	2.99	19.62	4.80

**Median**	9.67	3.44	10.56	3.10
**IQR**	6.28	2.16	4.80	2.03

Ratios of axial second moments of area to torsion constants *I/K*

Sample	Control group (C)		Touch stimulus group (TS)	
	Petiole	Transition zone	Petiole	Transition zone
	[-]	[-]	[-]	[-]
1	0.52	0.99	0.88	1.09
2	0.40	0.67	0.90	3.68
3	0.65	0.35	0.43	6.00
4	0.55	0.72	0.63	0.76
5	0.44	0.72	0.74	0.97
6	0.40	0.59	0.59	0.81
7	0.76	0.95	0.68	0.53
8	0.44	0.47	0.44	0.73
9	0.55	0.27	0.59	0.62
10	0.40	0.58	0.51	3.15
11	0.78	1.13	0.53	4.26
12	0.64	0.72	0.73	2.61
13	0.52	3.22	0.60	0.73
14	0.48	0.76	0.59	0.66
15	0.57	0.81	0.44	1.02
16	0.51	0.72	0.61	1.16
17	0.66	0.43	0.73	0.88
18	0.76	0.66	0.95	0.39
19	0.52	0.79	0.44	0.54
20	0.54	0.91	0.63	0.64
**Median**	0.53	0.72	0.60	0.84
**IQR**	0.17	0.25	0.20	0.86

Sample	Wind stimulus group (WS)		Touch and wind stimulus group (TWS)	
	Petiole	Transition zone	Petiole	Transition zone
	[-]	[-]	[-]	[-]
1	0.57	0.92	0.85	0.78
2	0.51	0.89	0.48	0.73
3	0.41	0.70	0.60	1.65
4	0.59	0.62	0.78	0.84
5	0.48	1.04	0.52	0.66
6	0.51	1.05	0.78	2.67
7	0.87	1.50	0.38	0.92
8	0.49	0.51	0.49	1.12
9	0.65	1.10	0.59	0.61
10	0.58	1.34	0.56	0.67
11	0.47	0.73	0.55	0.76
12	0.48	1.42	0.59	1.23
13	0.58	0.49	0.43	0.76
14	0.53	0.89	0.54	0.53
15	0.51	0.56	0.68	0.77
16	0.59	0.84	0.61	0.44
17	0.54	0.62	0.60	0.55
18	0.51	0.62	0.79	2.26
19	0.60	1.02	0.48	0.85

**Median**	0.53	0.89	0.59	0.77
**IQR**	0.08	0.43	0.13	0.36

Sample	Control group (C)		Touch stimulus group (TS)	
	Petiole	Transition zone	Petiole	Transition zone
	[mm^4]	[mm^4]	[mm^4]	[mm^4]
1	10.13	4.14	15.36	4.22
2	17.04	5.72	21.36	13.40
3	9.41	1.55	11.79	26.02
4	10.28	4.61	9.82	3.37
5	10.96	6.18	18.94	5.70
6	13.21	3.84	8.12	4.92
7	18.57	8.49	13.14	5.20
8	15.61	4.50	15.42	4.83
9	15.42	9.12	11.52	3.73
10	19.59	5.86	23.31	33.68
11	24.74	7.35	8.73	15.57
12	9.96	4.05	26.04	13.68
13	13.94	14.09	14.07	6.70
14	15.97	8.99	15.65	6.78
15	16.79	8.69	18.50	7.05
16	13.69	7.71	13.11	4.97
17	17.12	4.64	17.30	7.82
18	22.60	4.55	22.37	6.20
19	14.92	5.23	36.66	10.67
20	11.82	8.64	7.89	2.99
**Median**	15.17	5.79	15.39	6.45
**IQR**	5.46	3.99	7.82	6.45

Sample	Wind stimulus group (WS)		Touch and wind stimulus group (TWS)	
	Petiole	Transition zone	Petiole	Transition zone
	[mm^4]	[mm^4]	[mm^4]	[mm^4]
1	5.32	3.77	11.27	3.71
2	7.35	7.04	15.03	6.07
3	13.21	5.59	10.41	6.01
4	27.24	6.29	16.94	5.44
5	17.48	7.77	11.07	4.18
6	9.01	4.24	16.54	10.80
7	11.69	5.19	10.17	4.37
8	12.96	5.82	22.63	7.93
9	17.28	5.54	7.02	2.26
10	11.20	5.75	20.45	6.44
11	19.41	7.61	21.84	6.02
12	6.91	4.95	12.61	4.65
13	14.20	5.76	12.42	5.06
14	10.58	4.21	12.40	4.47
15	11.02	5.74	19.80	6.26
16	10.57	5.07	12.49	2.53
17	11.61	4.09	14.72	6.82
18	9.91	5.26	12.42	7.06
19	17.89	6.12	21.09	8.12

**Median**	11.61	5.59	12.61	6.01
**IQR**	5.50	0.96	6.54	2.21

Ratios of axial to polar second moments of area *I/J*

Sample	Control group (C)		Touch stimulus group (TS)	
	Petiole	Transition zone	Petiole	Transition zone
	[-]	[-]	[-]	[-]
1	0.35	0.56	0.49	0.57
2	0.37	0.55	0.55	0.73
3	0.43	0.33	0.47	0.71
4	0.45	0.48	0.46	0.50
5	0.41	0.48	0.46	0.58
6	0.43	0.55	0.49	0.46
7	0.44	0.39	0.49	0.52
8	0.37	0.53	0.44	0.47
9	0.47	0.43	0.42	0.56
10	0.38	0.54	0.37	0.71
11	0.42	0.41	0.39	0.62
12	0.39	0.51	0.42	0.66
13	0.46	0.70	0.49	0.44
14	0.36	0.44	0.48	0.46
15	0.52	0.56	0.46	0.55
16	0.45	0.57	0.36	0.62
17	0.42	0.36	0.47	0.44
18	0.51	0.53	0.49	0.46
19	0.39	0.52	0.29	0.43
20	0.40	0.48	0.38	0.47
**Median**	0.42	0.51	0.46	0.54
**IQR**	0.06	0.11	0.07	0.16

Sample	Wind stimulus group (WS)		Touch and wind stimulus group (TWS)	
	Petiole	Transition zone	Petiole	Transition zone
	[-]	[-]	[-]	[-]
1	0.43	0.46	0.55	0.48
2	0.48	0.52	0.36	0.44
3	0.41	0.43	0.47	0.62
4	0.31	0.51	0.52	0.48
5	0.43	0.47	0.42	0.52
6	0.38	0.55	0.49	0.75
7	0.47	0.61	0.47	0.50
8	0.37	0.42	0.40	0.55
9	0.52	0.61	0.40	0.43
10	0.39	0.60	0.36	0.49
11	0.45	0.48	0.41	0.52
12	0.39	0.58	0.41	0.50
13	0.51	0.41	0.46	0.46
14	0.46	0.51	0.44	0.54
15	0.42	0.50	0.52	0.58
16	0.50	0.52	0.38	0.38
17	0.45	0.57	0.43	0.45
18	0.50	0.54	0.45	0.67
19	0.47	0.50	0.45	0.50

**Median**	0.45	0.51	0.44	0.50
**IQR**	0.07	0.08	0.06	0.08

Sample	Control group (C)	Touch stimulus group (TS)	Wind stimulus group (WS)	Touch and wind stimulus group (TWS)
	[-]	[-]	[-]	[-]
1	1.04	1.04	1.15	1.20
2	1.79	1.06	0.89	1.15
3	0.96	1.45	1.83	0.90
4	0.69	1.69	1.22	2.00
5	0.89	0.86	2.35	1.31
6	1.10	1.39	0.97	0.87
7	1.01	1.30	0.79	2.32
8	1.92	1.53	1.20	1.03
9	1.39	0.89	1.00	0.77
10	1.12	1.34	1.52	1.04
11	1.08	0.93	2.60	1.29
12	0.88	0.94	1.10	1.64
13	1.08	1.15	1.45	2.06
14	1.08	1.06	0.70	1.17
15	1.12	1.81	0.84	1.67
16	0.57	1.05	1.01	0.82
17	1.06	0.98	0.82	1.57
18	0.92	1.73	1.86	1.03
19	0.92	0.90	1.25	0.91
20	0.99	1.26	-	-
**Median**	1.05	1.11	1.15	1.17
**IQR**	0.18	0.43	0.56	0.63

Sample	Control group (C)		Touch stimulus group (TS)	
	Petiole	Transition zone	Petiole	Transition zone
	[mm]	[mm]	[mm]	[mm]
1	2.89	2.21	3.05	2.18
2	3.56	2.62	3.40	2.28
3	2.83	1.96	3.38	2.37
4	3.04	2.36	2.91	2.17
5	3.20	2.55	3.31	2.42
6	3.45	2.45	2.87	2.31
7	3.24	2.44	3.13	2.68
8	3.41	2.68	3.53	2.37
9	3.40	3.47	3.02	2.42
10	3.72	2.74	3.63	2.97
11	3.42	2.28	2.84	2.19
12	2.81	2.32	3.53	2.44
13	3.35	2.36	3.29	2.53
14	3.32	2.70	3.37	2.63
15	3.52	2.79	3.73	2.49
16	3.34	2.81	2.98	2.28
17	3.23	2.50	3.26	2.52
18	3.52	2.47	3.30	2.95
19	3.27	2.43	3.97	3.06
20	3.08	2.60	2.63	2.17
**Median**	3.33	2.49	3.30	2.42
**IQR**	0.26	0.33	0.42	0.28

Sample	Wind stimulus group (WS)		Touch and wind stimulus group (TWS)	
	Petiole	Transition zone	Petiole	Transition zone
	[mm]	[mm]	[mm]	[mm]
1	2.53	2.09	2.94	2.19
2	2.90	2.54	3.28	2.47
3	3.41	2.43	3.02	2.19
4	3.48	2.69	3.28	2.37
5	3.55	2.45	3.09	2.42
6	2.89	2.18	3.21	2.35
7	2.83	2.16	3.36	2.21
8	3.16	2.64	3.70	2.51
9	3.45	2.37	2.65	2.01
10	2.96	2.26	3.40	2.63
11	3.69	2.67	3.58	2.54
12	2.75	2.12	3.08	2.10
13	3.36	2.65	3.42	2.36
14	3.11	2.23	3.19	2.60
15	3.11	2.69	3.52	2.62
16	3.10	2.38	2.99	2.16
17	3.15	2.48	3.22	2.74
18	3.15	2.61	2.92	2.15
19	3.45	2.35	3.76	2.64

**Median**	3.15	2.43	3.22	2.37
**IQR**	0.49	0.38	0.36	0.38

Sample	Control group (C)		Touch stimulus group (TS)	
	Petiole	Transition zone	Petiole	Transition zone
	[N]	[N]	[N]	[N]
1	193.58	19.28	241.42	22.48
2	321.86	36.43	210.37	34.09
3	226.28	28.59	375.39	18.53
4	294.08	69.75	297.43	53.63
5	250.79	40.51	155.52	22.74
6	86.24	36.20	300.60	27.25
7	244.01	70.77	265.25	34.89
8	308.22	71.52	488.44	50.45
9	104.64	15.46	327.34	35.48
10	277.88	43.12	478.46	67.13
11	137.07	46.85	296.54	37.57
12	151.87	15.01	418.36	72.70
13	107.11	41.41	443.03	54.04
14	201.84	53.72	412.88	54.54
15	446.23	66.44	187.73	49.67
16	236.21	32.49	288.86	37.04
17	277.86	76.87	371.15	45.55
18	290.34	20.72	176.55	75.13
19	307.10	47.37	215.26	112.57
20	173.37	34.49	237.61	43.45
**Median**	240.11	40.96	296.98	44.50
**IQR**	123.28	25.38	152.75	19.47

Sample	Wind stimulus group (WS)		Touch and wind stimulus group (TWS)	
	Petiole	Transition zone	Petiole	Transition zone
	[N]	[N]	[N]	[N]
1	246.38	68.38	293.50	24.32
2	344.99	46.70	180.18	32.01
3	292.53	32.62	71.75	49.29
4	419.20	56.14	348.43	40.14
5	377.84	65.27	124.90	36.28
6	33.96	46.32	276.75	122.59
7	164.42	60.49	149.16	31.82
8	108.61	64.38	602.86	46.76
9	160.67	29.01	196.87	28.82
10	362.58	97.84	388.61	59.45
11	257.82	37.73	540.41	52.75
12	209.02	42.20	423.43	26.05
13	345.38	42.84	406.31	63.67
14	304.97	37.86	207.65	51.23
15	334.12	57.78	425.24	33.03
16	220.26	46.27	177.99	58.51
17	374.21	61.02	107.90	75.25
18	218.47	24.35	258.22	24.06
19	260.61	37.49	509.10	68.14

**Median**	260.61	46.32	276.75	46.76
**IQR**	131.44	22.96	235.79	27.06

Sample	Control group (C)		Touch stimulus group (TS)	
	**Petiole**	**Transition zone**	**Petiole**	**Transition zone**
	[Nmm^2]	[Nmm^2]	[Nmm^2]	[Nmm^2]
1	117.49	8.92	234.86	10.62
2	292.44	19.09	240.37	38.64
3	148.14	4.88	324.30	30.80
4	192.41	28.42	219.72	19.47
5	174.29	19.52	152.75	12.56
6	71.18	15.50	197.44	11.10
7	227.39	33.26	227.84	16.55
8	281.08	32.10	434.55	20.74
9	90.08	7.99	241.24	15.42
10	257.59	22.81	486.23	117.64
11	154.17	21.12	168.36	38.00
12	102.95	6.12	457.87	73.49
13	95.80	45.43	402.88	24.68
14	158.87	28.74	363.02	26.38
15	446.11	44.25	261.87	29.12
16	182.27	20.84	210.35	20.71
17	240.18	24.78	338.28	22.71
18	330.99	9.49	212.44	34.46
19	245.79	22.66	228.87	64.12
20	124.95	19.85	132.28	14.02
**Median**	178.28	20.98	237.61	23.69
**IQR**	125.65	14.50	132.55	19.08

Sample	Wind stimulus group (WS)		Touch and wind stimulus group (TWS)	
	**Petiole**	**Transition zone**	**Petiole**	**Transition zone**
	[Nmm^2]	[Nmm^2]	[Nmm^2]	[Nmm^2]
1	116.54	24.47	244.59	8.96
2	200.72	26.08	143.07	14.13
3	243.59	13.36	50.78	30.21
4	422.19	28.50	418.34	17.96
5	377.25	34.38	89.25	15.56
6	20.68	21.71	258.61	130.75
7	119.62	34.76	116.00	13.23
8	77.51	26.03	604.69	29.13
9	160.45	16.96	104.76	7.58
10	261.82	57.62	332.71	29.25
11	301.06	19.94	540.59	26.95
12	111.34	21.70	348.41	11.32
13	322.16	17.14	375.58	26.19
14	215.39	15.95	160.80	23.25
15	235.60	29.15	487.04	19.86
16	170.56	23.11	124.54	14.70
17	267.68	28.14	94.65	35.31
18	176.15	11.94	203.45	17.79
19	264.50	18.58	498.30	39.19

**Median**	215.39	23.11	244.59	19.86
**IQR**	126.06	10.46	276.69	14.77

Sample	Control group (C)		Touch stimulus group (TS)	
	**Petiole**	**Transition zone**	**Petiole**	**Transition zone**
	[Nmm^2]	[Nmm^2]	[Nmm^2]	[Nmm^2]
1	25.50	65.46	24.06	37.97
2	58.53	92.92	57.94	59.27
3	30.51	66.53	39.30	33.37
4	44.73	29.32	44.71	11.72
5	41.37	28.96	66.93	28.94
6	41.27	20.15	44.32	27.43
7	52.10	136.78	44.04	56.29
8	41.97	116.56	83.70	22.31
9	81.22	26.40	34.30	99.49
10	51.99	22.77	67.88	122.86
11	70.34	79.05	21.45	23.67
12	27.19	8.16	88.73	96.24
13	34.97	65.39	39.54	53.55
14	42.37	29.21	60.47	42.56
15	54.60	131.78	64.02	23.88
16	54.04	19.31	34.85	49.20
17	54.13	35.36	54.37	25.44
18	49.46	97.07	95.93	22.44
19	39.02	34.13	79.15	75.07
20	34.92	17.84	25.21	19.97
**Median**	43.55	34.75	49.54	35.67
**IQR**	16.06	57.02	28.98	33.21

Sample	Wind stimulus group (WS)		Touch and wind stimulus group (TWS)	
	**Petiole**	**Transition zone**	**Petiole**	**Transition zone**
	[Nmm^2]	[Nmm^2]	[Nmm^2]	[Nmm^2]
1	24.65	69.26	33.81	73.94
2	45.01	61.40	29.82	18.91
3	29.29	22.13	28.11	72.01
4	64.02	119.76	47.69	19.78
5	81.97	20.97	31.52	17.57
6	21.90	47.04	43.41	104.38
7	27.40	30.79	53.86	20.40
8	44.99	46.12	82.12	155.66
9	47.21	93.47	17.27	27.76
10	37.25	15.07	72.52	92.20
11	72.17	31.03	61.10	33.25
12	21.95	90.26	32.23	13.09
13	31.19	44.96	62.71	23.03
14	28.86	58.49	45.23	29.66
15	54.59	54.08	47.72	13.15
16	26.00	26.52	44.41	53.63
17	47.22	47.36	74.78	27.50
18	32.45	20.21	26.37	28.80
19	39.07	31.45	67.68	33.53

**Median**	37.25	46.12	45.23	28.80
**IQR**	19.09	31.29	30.03	42.73

Sample	Control group (C)		Touch stimulus group (TS)	
	Petiole	Transition zone	Petiole	Transition zone
	[-]	[-]	[-]	[-]
1	4.61	0.14	9.76	0.28
2	5.00	0.21	4.15	0.65
3	4.86	0.07	8.25	0.92
4	4.30	0.97	4.91	1.66
5	4.21	0.67	2.28	0.43
6	1.72	0.77	4.46	0.40
7	4.36	0.24	5.17	0.29
8	6.70	0.28	5.19	0.93
9	1.11	0.30	7.03	0.16
10	4.95	1.00	7.16	0.96
11	2.19	0.27	7.85	1.61
12	3.79	0.75	5.16	0.76
13	2.74	0.69	10.19	0.46
14	3.75	0.98	6.00	0.62
15	8.17	0.34	4.09	1.22
16	3.37	1.08	6.04	0.42
17	4.44	0.70	6.22	0.89
18	6.69	0.10	2.21	1.54
19	6.30	0.66	2.89	0.85
20	3.58	1.11	5.25	0.70
**Median**	4.33	0.67	5.22	0.73
**IQR**	1.44	0.56	2.69	0.51

Sample	Wind stimulus group (WS)		Touch and wind stimulus group (TWS)	
	Petiole	Transition zone	Petiole	Transition zone
	[-]	[-]	[-]	[-]
1	4.73	0.35	7.23	0.12
2	4.46	0.42	4.80	0.75
3	8.32	0.60	1.81	0.42
4	6.59	0.24	8.77	0.91
5	4.60	1.64	2.83	0.89
6	0.94	0.46	5.96	1.25
7	4.37	1.13	2.15	0.65
8	1.72	0.56	7.36	0.19
9	3.40	0.18	6.07	0.27
10	7.03	3.82	4.59	0.32
11	4.17	0.64	8.85	0.81
12	5.07	0.24	10.81	0.86
13	10.33	0.38	5.99	1.14
14	7.46	0.27	3.56	0.78
15	4.32	0.54	10.21	1.51
16	6.56	0.87	2.80	0.27
17	5.67	0.59	1.27	1.28
18	5.43	0.59	7.71	0.62
19	6.77	0.59	7.36	1.17

**Median**	5.07	0.56	5.99	0.78
**IQR**	2.34	0.26	4.35	0.65

These results represent the *p*-values calculated by comparing the treatment groups for petioles and transition zones by post-hoc pairwise wilcoxon tests in R.

Material properties

Tensile elastic moduls E_t **Petiole** n = 20 (C, TS), n = 19 (WS, TWS)

**Group**	Control (C)	Touch stimulus (TS)	Touch and wind stimulus (TWS)
Touch stimulus (TS)	0.1005	NA	NA
Touch and wind stimulus (TWS)	0.8142	1.0000	NA
Wind stimulus (WS)	0.2504	1.0000	1.0000

Tensile elastic moduls E_t **Transition zone** n = 20 (C, TS), n = 19 (WS, TWS)

**Group**	Control (C)	Touch stimulus (TS)	Touch and wind stimulus (TWS)
Touch stimulus (TS)	1.0000	NA	NA
Touch and wind stimulus (TWS)	1.0000	1.0000	NA
Wind stimulus (WS)	0.6786	0.6786	1.0000

Torsional modulus G **Petiole** n = 20 (C, TS), n = 19 (WS, TWS)

**Group**	Control (C)	Touch stimulus (TS)	Touch and wind stimulus (TWS)
Touch stimulus (TS)	0.3574	NA	NA
Touch and wind stimulus (TWS)	1.0000	0.9867	NA
Wind stimulus (WS)	1.0000	0.5336	1.0000

Torsional modulus G **Transition zone** n = 20 (C, TS), n = 19 (WS, TWS)

**Group**	Control (C)	Touch stimulus (TS)	Touch and wind stimulus (TWS)
Touch stimulus (TS)	1.0000	NA	NA
Touch and wind stimulus (TWS)	1.0000	1.0000	NA
Wind stimulus (WS)	1.0000	1.0000	1.0000

Ratio of tensile elastic to torsional modulus E_t/G **Petiole** n = 20 (C, TS), n = 19 (WS, TWS)

**Group**	Control (C)	Touch stimulus (TS)	Touch and wind stimulus (TWS)
Touch stimulus (TS)	1.0000	NA	NA
Touch and wind stimulus (TWS)	1.0000	1.0000	NA
Wind stimulus (WS)	1.0000	1.0000	1.0000

Ratio of tensile elastic to torsional modulus E_t/G **Transition zone** *n* = 20 (C, TS), *n* = 19 (WS, TWS)

**Group**	Control (C)	Touch stimulus (TS)	Touch and wind stimulus (TWS)
Touch stimulus (TS)	1.0000	NA	NA
Touch and wind stimulus (TWS)	1.0000	1.0000	NA
Wind stimulus (WS)	1.0000	1.0000	1.0000

Geometrical properties

Cross-sectional area *A* **Petiole** *n* = 20 (C, TS), *n* = 19 (WS, TWS)

**Group**	Control (C)	Touch stimulus (TS)	Touch and wind stimulus (TWS)
Touch stimulus (TS)	1.0000	NA	NA
Touch and wind stimulus (TWS)	1.0000	1.0000	NA
Wind stimulus (WS)	0.7878	0.4738	0.7878

Cross-sectional area *A* **Transition zone**

**Group**	Control (C)	Touch stimulus (TS)	Touch and wind stimulus (TWS)
Touch stimulus (TS)	1.0000	NA	NA
Touch and wind stimulus (TWS)	1.0000	1.0000	NA
Wind stimulus (WS)	1.0000	1.0000	1.0000

Axial second moment of area *I* **Petiole** *n* = 20 (C, TS), *n* = 19 (WS, TWS)

**Group**	Control (C)	Touch stimulus (TS)	Touch and wind stimulus (TWS)
Touch stimulus (TS)	1.0000	NA	NA
Touch and wind stimulus (TWS)	1.0000	1.0000	NA
Wind stimulus (WS)	1.0000	0.5357	0.5784

Axial second moment of area *I* **Transition zone** *n* = 20 (C, TS), *n* = 19 (WS, TWS)

**Group**	Control (C)	Touch stimulus (TS)	Touch and wind stimulus (TWS)
Touch stimulus (TS)	1.0000	NA	NA
Touch and wind stimulus (TWS)	1.0000	1.0000	NA
Wind stimulus (WS)	1.0000	1.0000	1.0000

Torsion constant *K* **Petiole** *n* = 20 (C, TS), *n* = 19 (WS, TWS)

**Group**	Control (C)	Touch stimulus (TS)	Touch and wind stimulus (TWS)
Touch stimulus (TS)	1.0000	NA	NA
Touch and wind stimulus (TWS)	1.0000	1.0000	NA
Wind stimulus (WS)	1.0000	1.0000	1.0000

Torsion constant *K* **Transition zone** *n* = 20 (C, TS), *n* = 19 (WS, TWS)

**Group**	Control (C)	Touch stimulus (TS)	Touch and wind stimulus (TWS)
Touch stimulus (TS)	1.0000	NA	NA
Touch and wind stimulus (TWS)	0.6039	1.0000	NA
Wind stimulus (WS)	1.0000	1.0000	1.0000

Ratio of axial second moment of area and torsion constant *I/K* **Petiole** *n* = 20 (C, TS), *n* = 19 (WS, TWS)

**Group**	Control	Touch stimulus	Touch and wind stimulus
Touch stimulus (TS)	0.5404	NA	NA
Touch and wind stimulus (TWS)	0.9372	0.9372	NA
Wind stimulus (WS)	0.9668	0.2273	0.9372

Ratio of axial second moment of area and torsion constant *I/K* **Transition zone** *n* = 20 (C, TS), *n* = 19 (WS, TWS)

**Group**	Control (C)	Touch stimulus (TS)	Touch and wind stimulus (TWS)
Touch stimulus (TS)	0.4859	NA	NA
Touch and wind stimulus (TWS)	0.9414	1.0000	NA
Wind stimulus (WS)	0.8304	1.0000	1.0000

Polar second moment of area *J* **Petiole** *n* = 20 (C, TS), *n* = 19 (WS, TWS)

**Group**	Control (C)	Touch stimulus (TS)	Touch and wind stimulus (TWS)
Touch stimulus (TS)	1.0000	NA	NA
Touch and wind stimulus (TWS)	1.0000	1.0000	NA
Wind stimulus (WS)	0.6335	0.5040	0.6335

Polar second moment of area *J* **Transition zone** *n* = 20 (C, TS), *n* = 19 (WS, TWS)

**Group**	Control (C)	Touch stimulus (TS)	Touch and wind stimulus (TWS)
Touch stimulus (TS)	1.0000	NA	NA
Touch and wind stimulus (TWS)	1.0000	1.0000	NA
Wind stimulus (WS)	1.0000	1.0000	1.0000

Ratio of axial to polar second moment of area *I/J* **Petiole** n = 20 (C, TS), n = 19 (WS, TWS)

**Group**	Control (C)	Touch stimulus (TS)	Touch and wind stimulus (TWS)
Touch stimulus (TS)	0.6485	NA	NA
Touch and wind stimulus (TWS)	1.0000	1.0000	NA
Wind stimulus (WS)	1.0000	1.0000	1.0000

Ratio of axial to polar second moment of area *I/J* **Transition zone** n = 20 (C, TS), n = 19 (WS, TWS)

**Group**	Control	Touch stimulus	Touch and wind stimulus
Touch stimulus (TS)	1.0000	NA	NA
Touch and wind stimulus (TWS)	1.0000	1.0000	NA
Wind stimulus (WS)	1.0000	1.0000	1.0000

Tapering mode *α* **Petiole** n = 20 (C, TS), n = 19 (WS, TWS)

**Group**	Control (C)	Touch stimulus (TS)	Touch and wind stimulus (TWS)
Touch stimulus (TS)	1.0000	NA	NA
Touch and wind stimulus (TWS)	0.9454	1.0000	NA
Wind stimulus (WS)	1.0000	1.0000	1.0000

Diameter *d* **Petiole** n = 20 (C, TS), n = 19 (WS, TWS)

**Group**	Control (C)	Touch stimulus (TS)	Touch and wind stimulus (TWS)
Touch stimulus (TS)	1.0000	NA	NA
Touch and wind stimulus (TWS)	1.0000	1.0000	NA
Wind stimulus (WS)	1.0000	1.0000	1.0000

Diameter *d* **Transition zone** n = 20 (C, TS), n = 19 (WS, TWS)

**Group**	Control (C)	Touch stimulus (TS)	Touch and wind stimulus (TWS)
Touch stimulus (TS)	1.0000	NA	NA
Touch and wind stimulus (TWS)	0.6039	1.0000	NA
Wind stimulus (WS)	1.0000	1.0000	1.0000

Aspect ratio of the diameters *AR* **Petiole** *n* = 20 (C, TS), *n* = 19 (WS, TWS)

**Group**	Control (C)	Touch stimulus (TS)	Touch and wind stimulus (TWS)
Touch stimulus (TS)	0.5971	NA	NA
Touch and wind stimulus (TWS)	0.5971	0.7919	NA
Wind stimulus (WS)	0.7606	0.0981	0.1478

Aspect ratio of the diameters *AR* **Transition zone** *n* = 20 (C, TS), *n* = 19 (WS, TWS)

**Group**	Control (C)	Touch stimulus (TS)	Touch and wind stimulus (TWS)
Touch stimulus (TS)	1.0000	NA	NA
Touch and wind stimulus (TWS)	1.0000	1.0000	NA
Wind stimulus (WS)	1.0000	1.0000	1.0000

Structural properties

Axial rigidity *EA* **Petiole** *n* = 20 (C, TS), *n* = 19 (WS, TWS)

**Group**	Control (C)	Touch stimulus (TS)	Touch and wind stimulus (TWS)
Touch stimulus (TS)	0.1699	NA	NA
Touch and wind stimulus (TWS)	1.0000	1.0000	NA
Wind stimulus (WS)	1.0000	1.0000	1.0000

Axial rigidity *EA* **Transition zone** *n* = 20 (C, TS), *n* = 19 (WS, TWS)

**Group**	Control (C)	Touch stimulus (TS)	Touch and wind stimulus (TWS)
Touch stimulus (TS)	1.0000	NA	NA
Touch and wind stimulus (TWS)	1.0000	1.0000	NA
Wind stimulus (WS)	1.0000	1.0000	1.0000

Flexural rigidity *EI* **Petiole** *n* = 20 (C, TS), *n* = 19 (WS, TWS)

**Group**	Control (C)	Touch stimulus (TS)	Touch and wind stimulus (TWS)
Touch stimulus (TS)	0.1086	NA	NA
Touch and wind stimulus (TWS)	1.0000	1.0000	NA
Wind stimulus (WS)	1.0000	0.7075	1.0000

Flexural rigidity *EI* **Transition zone** *n* = 20 (C, TS), *n* = 19 (WS, TWS)

**Group**	Control (C)	Touch stimulus (TS)	Touch and wind stimulus (TWS)
Touch stimulus (TS)	1.0000	NA	NA
Touch and wind stimulus (TWS)	1.0000	1.0000	NA
Wind stimulus (WS)	1.0000	1.0000	1.0000

Torsional rigidity *GK* **Petiole** *n* = 20 (C, TS), *n* = 19 (WS, TWS)

**Group**	Control (C)	Touch stimulus (TS)	Touch and wind stimulus (TWS)
Touch stimulus (TS)	1.0000	NA	NA
Touch and wind stimulus (TWS)	1.0000	1.0000	NA
Wind stimulus (WS)	0.8747	0.5357	0.8747

Torsional rigidity *GK* **Transition zone**

**Group**	Control (C)	Touch stimulus (TS)	Touch and wind stimulus (TWS)
Touch stimulus (TS)	1.0000	NA	NA
Touch and wind stimulus (TWS)	1.0000	1.0000	NA
Wind stimulus (WS)	1.0000	1.0000	1.0000

Twist-to-bend ratio *EI/GK* **Petiole** *n* = 20 (C, TS), *n* = 19 (WS, TWS)

**Group**	Control (C)	Touch stimulus (TS)	Touch and wind stimulus (TWS)
Touch stimulus (TS)	0.1699	NA	NA
Touch and wind stimulus (TWS)	0.4524	1.0000	NA
Wind stimulus (WS)	0.4464	1.0000	1.0000

Twist-to-bend ratio *EI/GK* **Petiole** *n* = 20 (C, TS), *n* = 19 (WS, TWS)

**Group**	Control (C)	Touch stimulus (TS)	Touch and wind stimulus (TWS)
Touch stimulus (TS)	0.9430	NA	NA
Touch and wind stimulus (TWS)	0.9430	1.0000	NA
Wind stimulus (WS)	1.0000	0.9430	0.9430

These results represent the *p*-values calculated by comparing the petiole and transition zones of each treatment group by post-hoc pairwise wilcoxon tests in R.

Material properties

Tensile elastic moduls E_t n = 20 (C, TS), n = 19 (WS, TWS)

Test group	*p*-Values
Control (C)	0.0000
Touch stimulus (TS)	0.0000
Wind stimulus (WS)	0.0000
Touch and wind stimulus (TWS)	0.0000

Torsional modulus G n = 20 (C, TS), n = 19 (WS, TWS)

Test group	*p*-Values
Control (C)	0.0037
Touch stimulus (TS)	0.0064
Wind stimulus (WS)	0.0002
Touch and wind stimulus (TWS)	0.0071

Ratio of tensile elastic to torsional modulus E_t/G n = 20 (C, TS), n = 19 (WS, TWS)

Test group	*p*-Values
Control (C)	0.0000
Touch stimulus (TS)	0.0000
Wind stimulus (WS)	0.0000
Touch and wind stimulus (TWS)	0.0000

Geometrical properties

Cross-sectional area A n = 20 (C, TS), n = 19 (WS, TWS)

Test group	*p*-Values
Control (C)	0.0000
Touch stimulus (TS)	0.0001
Wind stimulus (WS)	0.0000
Touch and wind stimulus (TWS)	0.0000

Axial second moment of area I n = 20 (C, TS), n = 19 (WS, TWS)

Test group	*p*-Values
Control (C)	0.0001
Touch stimulus (TS)	0.0696
Wind stimulus (WS)	0.0000
Touch and wind stimulus (TWS)	0.0000

Torsion constant *K* n = 20 (C, TS), n = 19 (WS, TWS)

Test group	p-Values
Control (C)	0.0172
Touch stimulus (TS)	0.0009
Wind stimulus (WS)	0.0000
Touch and wind stimulus (TWS)	0.0010

Ratio of axial second moment of area and torsion constant *I/K* n = 20 (C, TS), n = 19 (WS, TWS)

Test group	p-Values
Control (C)	0.0172
Touch stimulus (TS)	0.0009
Wind stimulus (WS)	0.0000
Touch and wind stimulus (TWS)	0.0010

Polar second moment of area *J* n = 20 (C, TS), n = 19 (WS, TWS)

Test group	p-Values
Control (C)	0.0000
Touch stimulus (TS)	0.0064
Wind stimulus (WS)	0.0000
Touch and wind stimulus (TWS)	0.0000

Ratio of axial to polar second moment of area *I/J* n = 20 (C, TS), n = 19 (WS, TWS)

Test group	p-Values
Control (C)	0.0037
Touch stimulus (TS)	0.0009
Wind stimulus (WS)	0.0003
Touch and wind stimulus (TWS)	0.0010

Diameter *d* n = 20 (C, TS), n = 19 (WS, TWS)

Test group	p-Values
Control (C)	0.0000
Touch stimulus (TS)	0.0000
Wind stimulus (WS)	0.0000
Touch and wind stimulus (TWS)	0.0000

Aspect ratio of the diameters *AR* n = 20 (C, TS), n = 19 (WS, TWS)

Test group	p-Values
Control (C)	0.0362
Touch stimulus (TS)	0.1140
Wind stimulus (WS)	0.0020
Touch and wind stimulus (TWS)	0.1956

Structural properties

Axial rigidity *EA* *n* = 20 (C, TS), *n* = 19 (WS, TWS)

Test group	*p*-Values
Control (C)	0.0000
Touch stimulus (TS)	0.0000
Wind stimulus (WS)	0.0000
Touch and wind stimulus (TWS)	0.0000

Flexural rigidity *EI* *n* = 20 (C, TS), *n* = 19 (WS, TWS)

Test group	*p*-Values
Control (C)	0.0000
Touch stimulus (TS)	0.0000
Wind stimulus (WS)	0.0000
Touch and wind stimulus (TWS)	0.0000

Torsional rigidity *GK* *n* = 20 (C, TS), *n* = 19 (WS, TWS)

Test group	*p*-Values
Control (C)	0.4304
Touch stimulus (TS)	0.3488
Wind stimulus (WS)	0.2579
Touch and wind stimulus (TWS)	0.6226

Twist-to-bend ratio *EI/GK* *n* = 20 (C, TS), *n* = 19 (WS, TWS)

Test group	*p*-Values
Control (C)	0.0000
Touch stimulus (TS)	0.0000
Wind stimulus (WS)	0.0000
Touch and wind stimulus (TWS)	0.0000

To verify the difference between the tensile elastic moduli measured for *Pilea peperomioides* in this study compared to the bending elastic moduli measured for *P. peperomioides* in the study by Langer *et al.* (2021), both bending and tensile tests were performed on 10 petioles and both bending and tensile elastic moduli were calculated.

The two-point bending tests were carried out in the linear elastic range of the petioles to avoid plastic deformation. Tensile tests were then carried out until failure of the petioles.

In addition, both older leaves, such as those in the study by Langer *et al.* (2021), and young leaves, such as in this study, were examined.

In order to exclude errors due to the consideration of the tapering mode, the calculations were carried out once with and once without the consideration of the tapering mode.

Calculation considering the tapering mode

Petiole sample	Leaf age	Bending elastic modulus E_b [MPa]	Tensile elastic modulus E_t [MPa]
1	young	77.76	31.09
2	young	135.43	54.88
3	young	119.62	50.67
4	young	127.94	58.61
5	young	82.89	49.24
6	old	126.01	42.88
7	old	136.41	34.65
8	old	124.17	43.21
9	old	200.20	55.03
10	old	129.11	48.80
**Median**		126.98	49.02
**IQR**		13.09	10.87

Calculation without considering the tapering mode

Petiole sample	Leaf age	Bending elastic modulus E_b [MPa]	Tensile elastic modulus E_t [MPa]
1	young	53.15	30.77
2	young	95.37	43.30
3	young	91.40	40.46
4	young	104.51	46.99
5	young	65.77	39.94
6	old	82.61	32.76
7	old	81.97	24.98
8	old	90.09	34.59
9	old	134.73	41.43
10	old	76.61	36.34
**Median**		86.35	38.14
**IQR**		16.43	7.98

Statistical significance was tested with an alpha value of 5% to determine significant differences and using the Wilcoxon test in R.
The results represent the *p* -values calculated by comparing young and old leaves and bending and tensile elastic moduli.

Comparison between young and old leaves

	**Elastic modulus**	***p*-value**
Tapering mode considered	Bending	0.150
	Tensile	0.420
Tapering mode not considered	Bending	1.000
	Tensile	0.220

No significant differences were found between young and old leaves in terms of the bending and tensile elastic moduli.

Comparison between bending and tensile elastic moduli

	***p*-value**
Tapering mode considered	0.002
Tapering mode not considered	0.002

Bending and tensile elastic moduli were significantly different.
Regardless of whether the calculations were made with or without the consideration of the tapering mode.

Therefore, neither the consideration of the tapering mode nor the age of the leaves is the reason for the significant differences between the bending and tensile elastic moduli.

References

Langer M, Kelbel MC, Speck T, Müller C, Speck O. 2021. Twist-to-bend ratios and safety factors of petioles having various geometries, sizes and shapes. *Frontiers in Plant Science* **12**, 765605.
