# Supplementary material for: Acclimation to wind loads and/or contact stimuli? A biomechanical study of peltate leaves of Pilea peperomioides
Source: J Exp Bot. 2021 Dec 10;73(4):1236–52. doi: 10.1093/jxb/erab541 (PMC8866637; doi:10.1093/jxb/erab541)
Supplement: erab541_suppl_supplementary_tables_S1-S2 [file erab541_suppl_supplementary_tables_s1-s2.pdf]

Supplementary Table S1. Descriptive statistics of the variables measured and calculated for the leaf parts of *Pilea peperomioides*, namely petioles (P) and petiole-lamina transition zones (TZ), for each treatment group. The treatment groups are as follows: control (C), touch stimulus (TS), wind stimulus (WS) and combined touch and wind stimulus (TWS). Descriptions of material, geometrical and structural properties are given in Table 1. The sample size for the C and TS group is n = 20 and for the WS and TWS group n = 19.

Variable	Leaf part	C		TS		WS		TWS	
		Median	(IQR)	Median	(IQR)	Median	(IQR)	Median	(IQR)
**Material properties**									
E_t	TZ	7.07	(4.55)	7.05	(3.86)	8.75	(3.83)	7.71	(3.85)
[MPa]	P	31.30	(12.86)	44.75	(18.51)	44.39	(18.45)	36.91	(23.83)
G	TZ	9.76	(18.59)	10.10	(8.20)	10.54	(14.89)	8.10	(15.88)
[MPa]	P	5.41	(1.55)	6.36	(2.32)	5.49	(3.10)	6.06	(1.92)
E_t / G	TZ	0.89	(1.04)	0.53	(0.82)	0.78	(0.55)	0.70	(0.90)
[-]	P	5.70	(3.00)	6.48	(3.05)	7.24	(2.93)	6.54	(4.41)
**Geometrical properties**									
A	TZ	6.01	(1.89)	6.33	(2.74)	5.81	(0.67)	5.99	(1.13)
[mm^2]	P	9.62	(1.77)	9.64	(2.45)	8.51	(1.91)	8.80	(2.09)
I	TZ	3.00	(1.62)	3.09	(3.36)	2.84	(0.89)	2.65	(1.40)
[mm^4]	P	5.99	(2.59)	7.17	(3.85)	5.20	(2.87)	5.73	(3.49)
K	TZ	3.76	(2.08)	3.39	(1.54)	3.44	(2.16)	3.10	(2.03)
[mm^4]	P	12.03	(3.63)	11.58	(5.60)	9.67	(6.28)	10.56	(4.80)
I/K	TZ	0.72	(0.25)	0.84	(0.86)	0.89	(0.43)	0.77	(0.36)
[-]	P	0.53	(0.17)	0.60	(0.20)	0.53	(0.08)	0.59	(0.13)

α [-]	P	1.05	(0.18)	1.11	(0.43)	1.15	(0.56)	1.17	(0.63)
**Structural properties**									
EA	TZ	40.96	(25.38)	44.50	(19.47)	46.32	(22.96)	46.76	(27.06)
[N]	P	240.11	(123.28)	296.98	(152.75)	260.61	(131.44)	276.75	(235.79)
EI	TZ	20.98	(14.50)	23.69	(19.08)	23.11	(10.46)	19.86	(14.77)
[Nmm^2]	P	178.28	(125.65)	237.61	(132.55)	215.39	(126.06)	244.59	(276.69)
GK	TZ	34.75	(57.02)	35.67	(33.21)	46.12	(31.29)	28.80	(42.73)
[Nmm^2]	P	43.55	(16.06)	49.54	(28.98)	37.25	(19.09)	45.23	(30.03)
EI / GK	TZ	0.67	(0.56)	0.73	(0.51)	0.56	(0.26)	0.78	(0.65)
[-]	P	4.33	(1.44)	5.22	(2.69)	5.07	(2.34)	5.99	(4.35)

Supplementary Table S2. Ratios of median values of petiole-lamina transition zone to petiole for each variable and for each treatment group. The statistical significance level between transition zone and petiole is given for each treatment group. The treatment groups are as follows: control (C), touch stimulus (TS), wind stimulus (WS) and touch and wind stimulus (TWS). Descriptions of material, geometrical and structural properties are given in Table 1. The sample size for the C and TS group is n = 20 and for the WS and TWS group n = 19. The asterisks for the significance level are assigned to the following p-values: n.s. = not significant p-value ≥ 0.05, * 0.05 > p-value ≥ 0.01, ** 0.01 > p-value ≥ 0.001, *** 0.001 > p-value.

	Ratio of the median values of transition zone to petiole per treatment group				
**Variable**	**C**	**TS**	**WS**	**TWS**	**Significance level**
**Material properties**					
E_t	0.23	0.16	0.20	0.21	***
G	1.80	1.59	1.92	1.34	** / ***
E_t / G	0.16	0.08	0.11	0.11	***
**Geometrical properties**					
A	0.62	0.66	0.68	0.68	***
I	0.50	0.43	0.55	0.46	*** (TS = n.s.)
K	0.31	0.29	0.36	0.29	***
I/K	1.35	1.39	1.69	1.32	* / ** / ***
**Structural properties**					
EA	0.17	0.15	0.18	0.17	***
EI	0.12	0.10	0.11	0.08	***
GK	0.80	0.72	1.24	0.64	n.s.
EI / GK	0.15	0.14	0.11	0.13	***
